# Life-course social disparities in body mass index trajectories across adulthood: cohort study evidence from China health and nutrition survey

**DOI:** 10.1186/s12889-023-16881-4

**Published:** 2023-10-09

**Authors:** Yusong Dang, Xinyu Duan, Peixi Rong, Mingxin Yan, Yaling Zhao, Baibing Mi, Jing Zhou, Yulong Chen, Duolao Wang, Leilei Pei

**Affiliations:** 1 Department of Epidemiology and Health Statistics, School of Public Health, Xi’an Jiaotong University Health Science Center, Xi’an, 710061 Shaanxi P.R. China; 2 Department of Pediatrics, The Second Affiliated Hospitical of Xi’an Jiaotong University, Xi’an, 710004 Shaanxi P.R. China; 3 Institute of Basic and Translational Medicine, Shaanxi Key Laboratory of Ischemic Cardiovascular Disease, Shaanxi Key Laboratory of Brain Disorders, Xi’an Medical University, Xi’an, 710021 Shaanxi P.R. China; 4 Biostatistics Unit, Department of Clinical Sciences, Liverpool School of Tropical Medicine, Pembroke Place, Liverpool, L3 5QA UK; 5 Department of Neurology, Guangdong Key Laboratory of Age-Related Cardiac and Cerebral Diseases, Affiliated Hospital of Guangdong Medical University, Zhanjiang, China

**Keywords:** Body mass index trajectories, Life-course socioeconomic position, The growth mixture model

## Abstract

**Background:**

The social disparities in obesity may originate in early life or in adulthood, and the associations of socioeconomic position (SEP) with obesity could alter over time. It is unclear how lifetime-specific and life-course SEP influence adult obesity development in China.

**Methods:**

Based on the China Health and Nutrition Survey (CHNS), three SEP-related indicators, including the father’s occupational position and the participant’s education and occupational position, were obtained. The life-course socioeconomic changes and a cumulative SEP score were established to represent the life-course SEP of the participants in the study. The growth mixture modeling was used to identify BMI trajectories in adulthood. Multinomial logistic regression was adopted to assess the associations between SEP and adult BMI trajectories.

**Results:**

A total of 3,138 participants were included in the study. A positive correlation was found between the paternal occupational position, the participants’ occupational position, education, and obesity in males, whereas an inverse correlation was observed among females. Males who experienced social upward mobility or remained stable high SEP during the follow-up had 2.31 and 2.52-fold risks of progressive obesity compared to those with a stable-low SEP. Among females, stable high SEP in both childhood and adulthood was associated with lower risks of progressive obesity (OR = 0.63, 95% CI: 0.43–0.94). Higher risks of obesity were associated with the life-course cumulative SEP score among males, while the opposite relationship was observed among females.

**Conclusions:**

The associations between life-course SEP and BMI development trajectories differed significantly by gender. Special emphasis should be placed on males experiencing upward and stable high socioeconomic change.

**Supplementary Information:**

The online version contains supplementary material available at 10.1186/s12889-023-16881-4.

## Introduction

Obesity has emerged as a significant a public health concern worldwide owing to its strong association with various adverse physical and mental health outcomes, including hypertension, diabetes, cancer, depression and mortality [1–3]. The current obesity and overweight epidemics are influenced by personal and environmental factors, such as genes, diet culture, behavioral patterns and so on [4]. Notably, socioeconomic positions (SEP), encompassing education, income, and occupation, plays a crucial role in obesity development. Education is frequently used as a common measure of SEP, capturing the long-term effects of early life environments on adult health. Occupation can reflect a person’s social standing, income and intellect, thereby characterizing adult SEP [5]. Prior research has consistently found an inverse correlation between SEP, characterized by occupational social class and education, and obesity in high-income countries [6]. For instance, in the United Kingdom, adults with low occupational social class and educational attainment are observed to experience a higher risk for obesity [7]. However, the results are different in lower-income countries. A review by Dinsa and Goryakin concluded that more affluent people and better-educated individuals are more likely to be obese in low- and middle-income countries [8], suggesting the existence of socioeconomic disparities in obesity.

It is important to note that SEP (e.g. education, occupation) is not static but rather develops during the life course, and thus examining SEP at a certain moment in life does not reflect the temporal nature of this association or explain how it changes over time [9]. Several models have been proposed to study the impact of SEP at different life stage on diseases in adulthood. The social mobility model emphasizes the dynamic nature of SEP and incorporates the trajectory of socioeconomic mobility over one’s lifetime in determining disease risk [10]. The accumulation of risk model highlights the accumulated effects of exposure to adverse SEP during the life course [11]. The sensitive-periods model acknowledges that adverse socioeconomic exposures at certain developmental periods could exert a critical impact on adult health status [11]. For example, a cohort study in Brazil found the upwardly mobile income from childhood to adulthood protected against adult adiposity compared with remaining in the low-income group [12]. A Spanish study showed that the prevalence of overweight was 1.46 times higher among residents experiencing the accumulation of low educational attainment during 20 years compared with residents with accumulative high educational attainment [13]. In the United States, a longitudinal 19-year study suggested that higher parental education in childhood protected against weight gain over adulthood, and the protective effects of parental education were stronger than adult education [14]. Therefore, exploring the individual cumulative and dynamic nature of SEP associated with adult obesity may help us understand how social inequities in health develop and are maintained.

China is a rapidly growing economy, and recent studies suggest that the prevalence of overweight and obesity among Chinese adults aged ≥ 18 has increased to 34.3% and 16.4%, respectively, in 2018, making it the country with the largest number of obese individuals [15]. However, studies on the effects of cumulative and dynamic exposure to SEP over the life course on adult BMI development in China are scarce, and it is unclear whether SEP in early life has an impact on adult BMI development. Therefore, investigating the relationship between life-course SEP and the risk of adult obesity and overweight in China is essential to help the Chinese government establish public health priorities.

In this study, we used the China Health and Nutrition Survey (CHNS), a 20-year longitudinal survey, to obtain three SEP-related indicators, including the father’s occupation, the participant’s educational attainment, and the participants’ occupation. Using these indicators, we constructed two indices, life-course socioeconomic changes and the cumulative SEP score, to represent the life-course SEP of participants in the study and assess the influence of lifetime-specific and life-course SEP on adult obesity development. Our main objectives were (1) to investigate the latent effects of early-life socioeconomic circumstances on adult obesity, and (2) to determine the impact of individual SEP mobility and the cumulative life-course SEP during the life course on the BMI development trajectory in adulthood.

## Methods

### Study population

The study population was based on data from the China Health and Nutrition Survey (CHNS), an ongoing cohort study that aims to investigate the impact of economic, sociological, and demographic factors on the health and nutritional status of China’s population. The survey began in 1989 and subsequently has been tracked in 1989, 1991, 1993, 1997, 2000, 2004, 2006, 2009, 2011 and 2015. A multistage random cluster sampling method was used to select participants from 239 communities in nine provinces of China. The survey was approved by institutional review boards at the University of North Carolina, Chapel Hill (Chapel Hill, NC) and the China Center for Disease Control and Prevention (Beijing, China), and all participant provided written informed consent. A more detailed description of the design and procedures of CHNS has been described elsewhere [16].

**Fig. 1:**
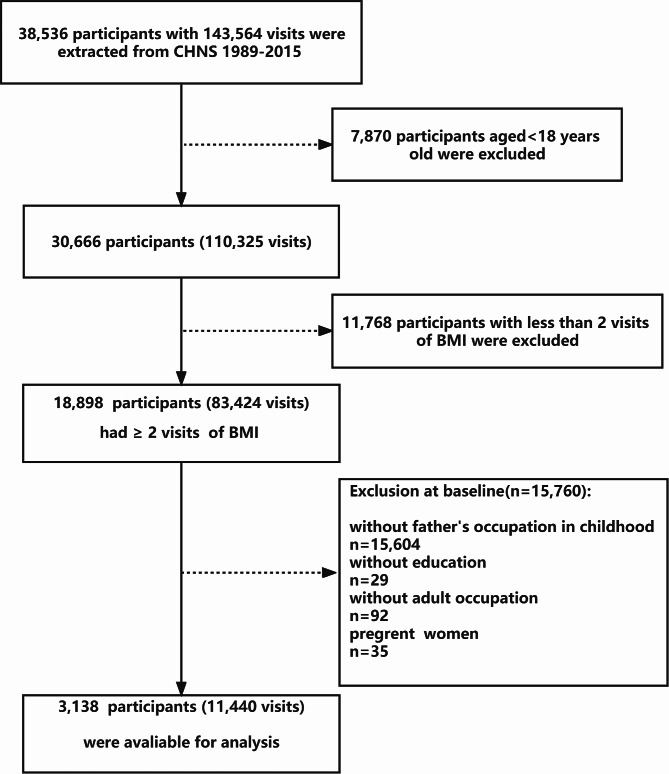
Flow diagram for cohort selection and censure

For our analysis, longitudinal data from ten waves of the CHNS between 1989 and 2015 were used. The flow diagram of the study cohort is summarized in Fig. 1. Initially, a total of 38,536 participants were extracted from the original surveys, but 35,398 participants were excluded for various reasons, such as being under 18 years old (n = 7,870), having less than two BMI measurement in adulthood (n = 11,768), missing data on the father’s occupation (n = 15,604), missing data on participant’s education (n = 29) and occupation (n = 92) in adulthood, and being pregnant females (n = 35). Finally, a total of 3,138 participants with 11,440 visits were included in the study.

### Outcome assessment

Weight and height measurements were conducted in a standardized manner using a balance-beam scale with an accuracy of 0.1 kg and a portable stadiometer with an accuracy of 0.1 cm. BMI was calculated as weight (in kilograms) divided by the square of the height (in meters). Two or more measurements per subject were obtained in the China Health and Nutrition Survey (CHNS) between 1989 and 2015, and the distribution of ages at each measurement wave can be found in Additional file 1: Table 3. The definition of overweight and obesity in the Chinese population is lower than that in European or North American populations according to the Working Group on Obesity in China (WGOC) [17]. In the study, overweight and obesity were defined as 24 ≤ BMI < 28 kg/m^2^ and BMI ≥ 28 kg/m^2^, respectively [18]. The growth mixture model (GMM), a method that has been used to identify unobserved trajectory classes in epidemiological data, was utilized to identify trajectories of change in BMI. The trajectory membership of BMI was then treated as the main outcome variable in the study.

### Socioeconomic indicators

We collected three indicators at the baseline survey to represent individual socioeconomic position (SEP) during the life course. The father’s occupational position commonly indicates the SEP of the participants in childhood, while the participant’s occupation in adulthood is the most commonly used indicators of adult SEP [5]. Both were assessed retrospectively with the question “What is/was your father’s main job”, and “What is/was your main job”, respectively. According to the criteria of socioeconomic classification scheme [19], the father’s and participants’ occupation was categorized as high (social classes I–II), medium (social classes III–IV) and low (social class V) (Additional file 1: Table S1). The participant’s education was considered the main indicator of SEP in their own early life, especially in young adulthood [20]. Education was defined as the highest qualification in full-time education, and participants were asked the question “How many years of formal education did you have in school?” In the study, education was also grouped into high (≥ 12 years formal education), medium (8–11 years formal education), and low (< 8 years formal education).

Two additional indicators, life-course socioeconomic changes and the cumulative SEP score were established to represent life-course SEP of participants. The cumulative SEP score during the life course was derived using information on paternal occupational position, the participant’s education and occupational position. Each of the three individual SEP indicators was coded 0 to 2. These indicators were then combined, resulting in a total score ranging from 0 to 6, where higher values indicate a greater life-course advantage (Additional file 1: Table S2-1). Previous studies have confirmed the practicability of the accumulated SEP score during the life course [21–23].

The life-course socioeconomic changes from childhood to adulthood were computed using information on the father’s occupation and adult occupation, both of which were dichotomized as high (social class I-IV) or low (social class V) to derive this new variable [24]. Four possible combinations of socioeconomic changes across the life course were generated (Additional file 1: Table S2-2): high SEP in childhood and high SEP in adulthood (stable high, n = 1524), low SEP in childhood and high SEP in adulthood (upward, n = 581), high SEP in childhood and low SEP in adulthood (downward, n = 292), and low SEP in childhood and low SEP in adulthood (stable low, n = 741).

### Covariates

A set of covariates was considered in this study based on previous research and a priori knowledge about the data [25]. Sex (male or female), age (years), place of residence (urban or rural), smoking/drinking habits, total daily energy intake (TDEI), and occupational physical activity level (OPAL) were included as baseline covariates. Smoking status was determined by asking participants if they had ever smoked cigarettes (including hand-rolled or device-rolled), and participants were categorized as either yes (smoking currently) or no (not smoking currently). Drinking status was assessed by asking participants how often they drank beer, wine or other alcoholic beverage in the past years, and participants were categorized as either yes (drinking ≥ 1 per month last year) or no (drinking < 1 per month).

The total dietary energy intake (TDEI) was measured at both the household and individual levels. Household food consumption was measured by weighting food inventories for 3 consecutive days, while individual dietary intake was collected for 3 consecutive days for every household member. TDEI was calculated based on the China Food Composition Table [26], which was used to determine the energy content of each food item consumed by the individual. The caloric value of all food items was summed to calculate a daily total, and the average of total energy intake for 3 days was used. Occupational physical activity level (OPAL) was derived from the survey and classified into five categories: very light (sitting at work, such as office workers, watch repairers, etc.), light (standing at work, such as sales clerks, laboratory technicians, teachers, etc.), moderate (students, drivers, electricians, metal fabrication workers, etc.), heavy (farmers, dancers, steel workers, athletes, etc.) and very heavy (loaders, lumberjacks, miners, masons, etc.). OPAL was then reclassified into three categories: light OPAL (including very light and light OPAL activities), moderate OPAL (including moderate OPAL activities), and heavy OPAL (including heavy and very heavy OPAL activities).

In addition, smoking, drinking, occupational physical activity level, and total dietary energy intake were considered as time-varying variables in this prospective cohort studies. Participants were divided into four categories based on changes in smoking and drinking behavior during the follow-up period: (1) never smoking/drinking (neither smoking/drinking at baseline nor at the latest survey), (2) change to a smoker/drinker (smoking/drinking at the latest survey but not at baseline), (3) quit smoking/drinking (smoking/drinking at baseline but not at the latest survey), and (4) keep smoking/drinking (consistently smoking/drinking at baseline and the latest survey). Changes in OPAL and TDEI were calculated as the values at the current minus those at baseline, and OPAL was classified into three categories (increase, unchanged or decrease).

### Statistical analysis

Continuous variables were reported as the mean ± standard deviation (SD) if normally distributed and as median and interquartile range (IR) if not normally distributed. Categorical variables were reported as the frequency (percentages). Differences in baseline characteristics across different SEP indicators were compared using ANOVA, the Wilcoxon rank-sum test, and the chi-square test as appropriate.

The longitudinal development of BMI of participants between 18 and 81 years of age was analyzed using the growth mixture model (GMM) to investigate heterogeneity and identify distinct groups of individuals who shared similar underlying BMI trajectories. The growth mixture model specifications were determined through a series of steps, aiming to improve the Bayesian Information Criterion (BIC) when retaining theoretical plausibility, but entropy statistics were ignored because they do not measure model fit [27]. The age scale was centered at the mean of wave_2000 and wave_2004 (30.5 years) to aid numerical stability (Additional file 1: Table S3). Four age functions for the BMI trajectory shapes, including linear, quadratic, cubic and freely estimated polynomials, were assumed. The quadratic polynomial function provided the best fit for the data (Additional file 1: Table S4). Based on the quadratic polynomial models, we further relaxed some of the main default constraints in the Mplus implementation to achieve the best fitting model. First, heteroskedasticity in BMI residual variances was assumed across sweeps. We then allowed the different residual variances/errors across the classes and explored the better-fitting models [28]. The models were further extended to include a within-class auto-correlation structure for the residual variances/errors [29]. In the modeling process, we included sex as a covariate to adjust the pattern difference between sexes. The best-fitting model covered a quadratic polynomial function of age, within-class heteroscedastic errors, and a first-order autoregressive structure (AR1) to model auto-correlation. Second, the number of trajectories was chosen based on better goodness of fit ((i.e. lower Akaike Information Criteria (AIC), and Bayesian Information Criterion (BIC)), internal reliability (mean posterior probability > 0.7 for each latent class, reflecting an acceptable uncertainty of posterior classification), clinical plausibility, and interpretability (Additional file 1: Table S5). Further details about the implementation of the growth mixture model were provided in Additional file 1: Text. The GMM analysis in the study was in compliance with the Guidelines for Reporting on Latent Trajectory Studies (GRoLTS) Checklist (Additional file 1: Table S6).

The association between life-course socioeconomic position and adult BMI multi-class trajectories was explored using multinomial logistic regression model adjusting for other covariates. For categorical dependent variable with two or more unordered levels, separate logistic regressions for each category versus the baseline category were established. Adjusted odds ratios (OR) and 95% CI (confidence interval) were calculated for the different BMI trajectories. Five adjusted multinomial logistic regression models were established, adjusting for different covariates. Model 1 was the basic model adjusting for sex, age and place of residence. Model 2 was established based on Model 1 after adjustments for change in smoking and drinking. Model 3 was established based on Model 1 further adjusting for change in occupational physical activity level. Model 4 was established based on Model 1 with further adjustments for change in total daily energy intake. Model 5 was established adjusting for all covariates. Multicollinearity was checked using variance inflation factor (VIF), with > 4 representing no multicollinearity of the variables. All statistical analyses were performed using Stata version 12.0 and Mplus Version 8.3, and statistical significance was set at a 2-sided *p* < 0.05.

A series of sensitivity analysis were conducted to test the robustness of the results. First, we excluded participants who developed diabetes, hypertension, myocardial infarction, stroke or cancer at baseline to minimize the possible reverse causation from these diseases. Second, we imputed all missing covariates using multiple imputations by the chained equations method to handle missing covariates. We generated 20 imputed data sets, as the maximum missing amount of these variables was lower than 5%. The multinomial logistic regression analyses were repeated using each of the augmented data sets, and parameter estimates were averaged across the 20 sets. To explain the rationale for the inclusion of a specific set of confounders, we constructed a directed acyclic graph (DAG) based on prior knowledge [30, 31]. After selecting the model using the previously described DAG (Additional file 1—Figure 1), seven variables (age in years as continuous variable; sex, residence, change in smoking, change in drinking, change in OPAL and change in TDEI) were included in the final analysis. The potential mediation effect of lifestyle behaviors on the association between SEP and BMI trajectory patterns were assessed using the process ‘Marco’ in SPSS 23.0. The indirect effect and 95% CI were calculated by the bootstrapping procedure for n = 5000. Fourth, we integrated OPAL and TDEI into the BMI trajectory modeling using GMM and repeated all analyses. Fifth, taking into account that some young participants have not yet reached the highest level of education, the highest educational attainment was utilized to reanalyze the associations. Finally, considering only the completeness of SEP during adulthood while retaining the largest possible analytic sample, we test the stability of gender-specific associations between adult SEP and BMI trajectories.

## Results

### Long‑term BMI trajectories across adulthood

**Fig. 2:**
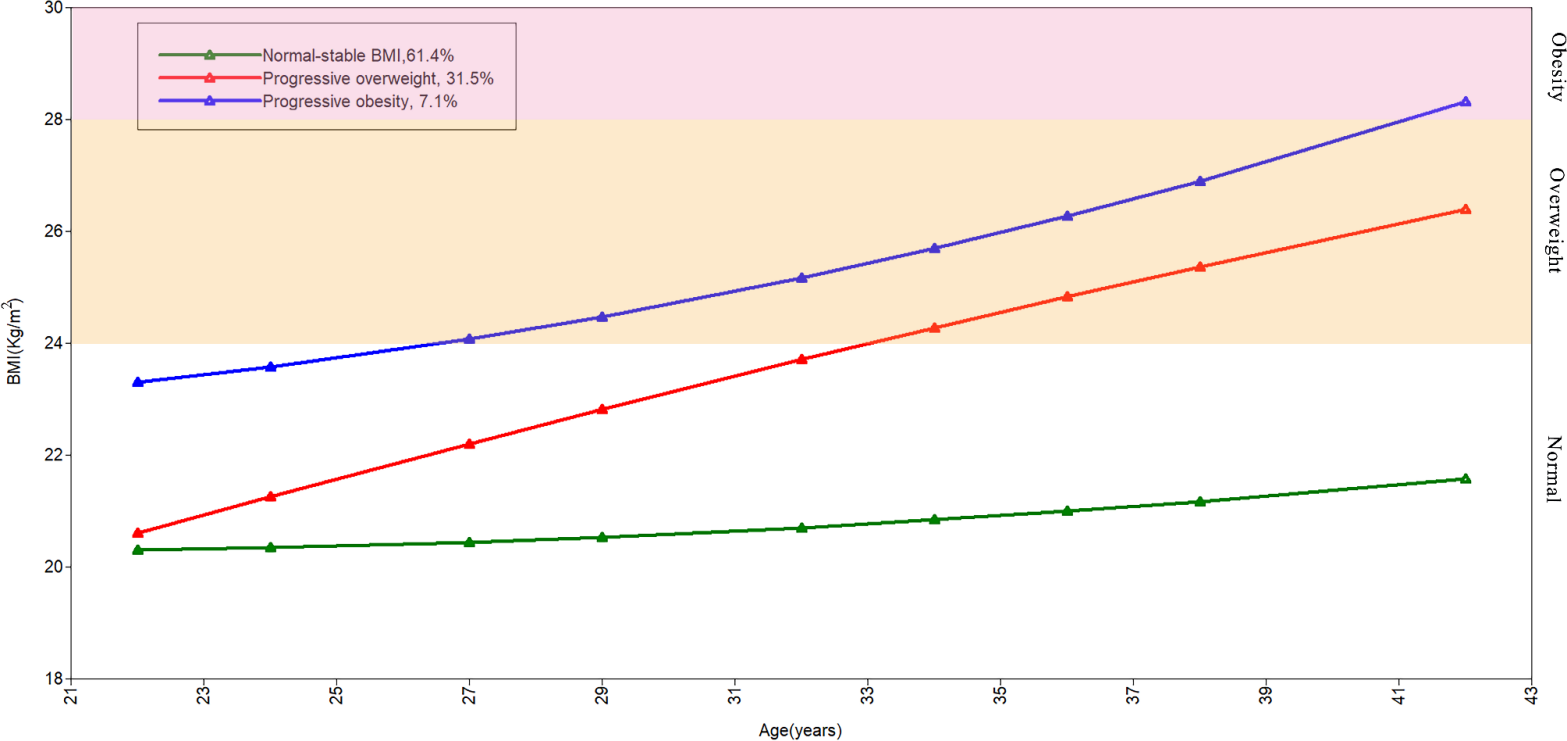
Three latent trajectories of BMI patterns

The growth mixture model with a quadratic polynomial function of age, within-class heteroscedastic errors, and a first-order autoregressive structure (AR1) was fitted to identify the best class solution for the BMI trajectory group (Additional file 1: Table S5). The model was fitted with 1–7 latent classes, and the best class solution was determined based on the lower BIC indicating better goodness of fit, the proportion of subjects classified in each group with a posterior probability > 0.7 and a minimum frequency of > 5%, clinical plausibility, and interpretability. Ultimately, the three-class model was selected as the optimal model for the BMI trajectory group, with three distinct classes: normal-stable BMI (N = 1927, 61.4%), progressive overweight (N = 988, 31.5%), and progressive obesity (N = 223, 7.1%) (Fig. 2). Specifically, the normal-stable BMI group had consistently normal BMI, while the progressive overweight group had continuously increasing BMI from normal to overweight, and the progressive obesity group had an elevated BMI from normal to obesity.

### Basic characteristics of the participants

Of the 3,138 participants included in the study (909 females), the median BMI and age were 20.7 (IQR: 3.1) and 23.0 (IQR:8.0) years, respectively, and the mean years of education was 9.6 ± 3.2 at baseline. Approximately 66.0% of participants lived in rural areas. Table 1 presents the baseline characteristics of participants across different SEP indicators. Participants with low SEP, including those with low father’s and own occupational position or adult education, were more likely to be younger and reside in rural areas. The TDEI, OPAL, smoking and drinking at baseline, change in smoking, change in drinking, change in OPAL, and change in TDEI were significantly different among the SEP patterns (father’s occupational position, adult occupational position or education).

**Table 1:** Characteristics of participants across different socioeconomic position indicators (n = 3138)^a^

Characteristics	Father’s occupational position			Adult occupational position			Adult education		
	Low	Medium	High	Low	Medium	High	Low	Medium	High
**Participants(n)**	1322(42.1)	955(30.4)	861(27.4)	1033(32.9)	1331(42.4)	774(24.7)	734(23.4)	1398(44.6)	1006(32.1)
**Baseline Age(years)**	19.0(7.0)	23.0(7.0)	23.0(6.0)	20.0(5.0)	24.0(8.0)	25.0(7.0)	22.0(9.0)	22.0(7.0)	24.0(6.0)
**Sex**									
Male	1008(76.2)	655(68.6)	566(65.7)	711(68.8)	996(74.8)	522(67.4)	508(69.2)	1024(73.2)	697(69.3)
Female	314(23.8)	300(31.4)	295(34.3)	322(31.2)	335(25.2)	252(32.6)	226(30.8)	374(26.8)	309(30.7)
**Residential areas**									
Urban	275(20.8)	374(39.2)	419(48.7)	190(18.4)	475(35.7)	403(52.1)	145(19.8)	428(30.6)	495(49.2)
Rural	1047(79.2)	581(60.8)	442(55.1)	843(81.6)	856(64.3)	371(47.9)	589(80.2)	970(69.4)	511(50.8)
^**BMI(Kg/m2)**^	20.6(2.8)	20.9(3.3)	20.8(3.4)	20.7(2.8)	20.8(3.2)	20.8(3.6)	20.7(2.8)	20.8(3.1)	20.8(3.4)
*Initial health-related behaviors at baseline*									
**Cigarette smoker**	501(37.9)	282(29.5)	232(26.9)	361(35.0)	440(33.1)	214(27.6)	272(37.1)	481(34.4)	262(26.0)
**Alcohol drinker**	514(38.9)	318(33.3)	310(36.0)	357(34.6)	471(35.4)	314(40.6)	271(36.9)	511(36.6)	360(35.8)
**OPAL**									
Light OPAL	176(13.5)	321(34.0)	344(40.6)	95(9.3)	354(27.2)	392(51.2)	91(12.5)	340(24.7)	410(41.5)
Moderate OPAL	320(24.6)	398(42.2)	368(43.4)	237(23.2)	550(42.2)	299(39.1)	176(24.2)	477(34.7)	433(43.9)
Heavy OPAL	803(61.8)	224(23.8)	135(15.9)	690(67.5)	398(30.6)	74(9.7)	459(63.2)	559(40.6)	144(14.6)
**TDEI (kcal)**	2622.0(952.2)	2355.0(872.2)	2400.5(925.8)	2678.0(972.0)	2424.8(865.2)	2298.9(903.3)	2654.0(882.2)	2484.2(973.0)	2352.4(848.3)
*Latest health-related behavior*									
**Cigarette smoker**	589(47.0)	371(29.6)	293(23.4)	424(41.0)	578(43.4)	251(32.4)	323(44.0)	582(41.6)	348(34.6)
**Alcohol drinker**	574(43.1)	404(30.3)	354(26.6)	405(39.2)	586(44.0)	341(44.1)	292(39.8)	614(43.9)	426(42.3)
**OPAL**									
Light OPAL	236(18.1)	437(46.3)	478(56.4)	114(11.2)	490(37.4)	547(71.4)	136(18.7)	430(31.2)	585(59.2)
Moderate OPAL	273(21.0)	315(33.4)	244(28.8)	159(15.6)	513(39.2)	160(20.9)	132(18.2)	429(31.2)	271(27.4)
Heavy OPAL	792(60.9)	192(20.3)	125(14.8)	743(73.1)	307(23.4)	59(7.7)	458(63.1)	518(37.6)	133(13.4)
**TDEI (kcal)**	2362.3(928.0)	2244.4(888.2)	2158.4(866.1)	2414.4(916.7)	2260.4(875.3)	2072.5(863.8)	2379.4(915.6)	2274.0(912.2)	2144.5(876.5)
*Change in time-varying health-related behavior*									
**Smoking**									
Never smoking	622(37.8)	507(30.8)	517(31.4)	526(50.9)	652(49.0)	468(60.5)	358(48.8)	699(50.0)	589(58.5)
Change to be a smoker	329(47.4)	195(28.1)	170(24.5)	244(23.6)	307(23.1)	143(18.5)	186(25.3)	302(21.6)	206(20.5)
Quit smoking	111(46.4)	77(32.2)	51(21.3)	83(8.0)	101(7.6)	55(7.1)	53(7.2)	117(8.4)	69(6.9)
Keep smoking	260(46.5)	176(31.5)	123(22.0)	180(17.4)	271(20.4)	108(14.0)	137(18.7)	280(20.0)	142(14.1)
**Drinking**									
Never drinking	603(45.6)	438(45.9)	407(47.3)	525(50.8)	598(44.9)	325(42.0)	373(50.8)	617(44.1)	458(45.5)
Change to be a drinker	355(26.9)	238(24.9)	218(25.3)	245(23.7)	352(26.4)	214(27.6)	177(24.1)	367(26.3)	267(26.5)
Quit drinking	145(11.0)	113(11.8)	100(11.6)	103(10.0)	147(11.0)	108(14.0)	69(9.4)	167(11.9)	122(12.1)
Keep drinking	219(16.6)	166(17.4)	136(15.8)	160(15.5)	234(17.6)	127(16.4)	115(15.7)	247(17.7)	159(15.8)
**OPAL**									
Decrease	277(21.5)	305(32.7)	296(35.4)	160(15.8)	420(32.6)	298(39.3)	153(21.3)	376(27.6)	349(27.6)
Remaining	770(59.8)	459(49.1)	384(45.9)	656(64.9)	609(47.3)	348(45.8)	439(61.1)	706(51.8)	468(48.0)
Increase	240(18.6)	170(18.2)	157(18.5)	195(19.3)	259(20.1)	113(14.9)	127(17.7)	282(20.7)	158(16.2)
**TDEI (kcal)**	147.2(953.3)	10.0(786.9)	38.2(785.9)	163.4(912.0)	58.0(918.4)	0.0(746.4)	130.9(966.7)	56.6(897.7)	23.13(793.4)

^a^ Data were expressed as numbers (percentages). Non-normally distributed data like baseline age was reported as median (IQR).

BMI: body mass index; OPAL: occupational physical activity level; TDEI: total daily energy intake

According to the three-class BMI trajectories (Table 2), higher total daily energy intake, unhealthy smoking and drinking, and lower heavy and moderate OPAL were prevalent among participants in the progressive obesity group. The progressive obesity group had higher father’s and own occupational position, but no significant difference was found in the participant’s education across the three trajectories. Most characteristics were significantly different between the analytic and excluded samples (Additional file 1: Table S7).

**Table 2:** Participant characteristics across BMI trajectory groups (n = 3138)^a^

Characteristics	BMI trajectory groups		
	Normal-stable BMI	Progressive overweight	Progressive obesity
**Participants(n)**	1927(61.4)	988(31.5)	223(7.1)
**Baseline age(years)**	23.0(7.0)	23.0(9.7)	24.0(7.0)
**Sex**			
Male	1261(65.4)	797(80.3)	171(76.7)
Female	666(34.6)	191(19.3)	52(23.3)
**Place of residence**			
Urban	669(34.7)	327(33.1)	72(32.3)
Rural	1258(65.3)	661(66.9)	151(67.7)
**BMI(Kg/m** ^**2**^ **)**	20.24(2.4)	21.8(3.8)	23.8(6.2)
*Socioeconomic positions*			
**Father’s occupational position**			
Low	809(42.0)	429(43.4)	84(37.7)
Medium	605(31.4)	279(28.2)	71(31.8)
High	513(26.6)	280(28.3)	68(30.5)
**Adult occupational position**			
Low	701(36.4)	270(27.3)	62(27.8)
Medium	785(40.7)	455(46.1)	91(40.8)
High	441(22.9)	263(26.6)	70(31.4)
**Adult education**			
Low	478(24.8)	216(21.9)	40(17.9)
Medium	849(44.1)	443(44.8)	106(47.5)
High	600(31.1)	329(33.3)	77(34.5)
*Change in time-varying health-related behavior*			
**Smoking**			
Never smoking	1081(56.1)	448(45.4)	116(52.0)
Change to be a smoker	408(21.2)	240(24.3)	46(20.6)
Quit smoking	126(6.5)	94(9.5)	19(8.5)
Keep smoking	312(16.2)	205(20.7)	42(18.8)
**Drinking**			
Never drinking	963(50.0)	394(40.0)	90(40.4)
Change to be a drinker	472(24.6)	285(28.8)	52(23.3)
Quit drinking	212(11.0)	109(11.0)	37(16.6)
Keep drinking	278(14.4)	199(20.1)	44(19.7)
**OPAL**			
Decrease	511(26.5)	296(30.0)	71(31.8)
Remaining	999(51.8)	498(50.4)	116(52.0)
Increase	362(18.8)	172(17.4)	33(14.8)
**TDEI (kcal)**	144.6(809.0)	127.6(968.7)	227.7(984.2)

^a^ Data were expressed as numbers (percentages). Non-normally distributed data like baseline age was reported as median (IQR). All univariate comparisons across subgroups were conducted using ANOVA, Chi-square test, and Kruskal-Wallis as appropriate. BMI: body mass index; OPAL: occupational physical activity level; TDEI: total daily energy intake

### The association of socioeconomic position in early life and adult life with BMI trajectories

As shown in Table 3, the participants with the high education (OR = 1.60, 95% CI:1.30,1.98) or occupational position (OR = 1.85, 95% CI:1.51, 2.26), were more likely to develop progressive obesity compared with those with the low education or occupational position. Participants with the high father’s occupational position (OR = 1.39, 95% CI:1.16,1.67) had a higher risk for progressive overweight than those with the low father’s occupational position. Apart from the father’s occupational position, similarly, high participant’s education (OR = 1.26, 95% CI:1.13, 1.41) and occupational position (OR = 1.64, 95% CI:1.46, 1.84) were associated with increased risk for progressive overweight after adjustment for all covariates. The results suggested that the ORs of adult occupational position were larger than that of education in adulthood. After stratifying by sex, the relationships between different SEP indicators at the baseline and BMI trajectories were further tested. Among the male participants, high SEP, such as through the father’s occupational position, adult occupation and education, were associated with a higher risk of progressive obesity and progressive overweight (Additional file 1: Table S8). Among the females, the high adult occupational position decreased the risks of progressive obesity (OR = 0.28, 95% CI:0.11, 0.75) and progressive overweight (OR = 0.59, 95% CI:0.37, 0.94), while the father’s occupational position and adult educational did not have a significant effect on the BMI trajectory groups (Additional file 1: Table S11).

**Table 3:** The association of socioeconomic position in early life and adult life with BMI trajectories^a^

	Progressive overweight vs. normal-stable BMI					
	Father’s occupational position^b^		Adult occupational position^b^		Adult education^b^	
	Low	High	Low	High	Low	High
	OR (95% CI)	OR (95% CI)	OR (95% CI)	OR (95% CI)	OR (95% CI)	OR (95% CI)
Model 1	1.00 (ref.)	1.13(0.93;1.37)	1.00(ref.)	1.57(1.26;1.97)	1.00(ref.)	1.23(1.02;1.53)
Model 2	1.00 (ref.)	1.13(0.93;1.37)	1.00(ref.)	1.58(1.41;1.77)	1.00(ref.)	1.23(1.10;1.37)
Model 3	1.00 (ref.)	1.12(0.92;1.38)	1.00(ref.)	1.58(1.41;1.78)	1.00(ref.)	1.24(1.12;1.39)
Model 4	1.00 (ref.)	1.16(0.95;1.41)	1.00(ref.)	1.61(1.44;1.81)	1.00(ref.)	1.24(1.11;1.39)
Model 5	1.00 (ref.)	1.16(0.95;1.42)	1.00(ref.)	1.64(1.46;1.84)	1.00(ref.)	1.26(1.13;1.41)
	Progressive obesity vs. normal-stable BMI					
	Father’s occupational position ^b^		Adult occupational position ^b^		Adult education ^b^	
	Low	High	Low	High	Low	High
	OR (95% CI)	OR (95% CI)	OR (95% CI)	OR (95% CI)	OR (95% CI)	OR (95% CI)
Model 1	1.00 (ref.)	1.41(1.18;1.68)	1.00(ref.)	1.92(1.31;2.82)	1.00(ref.)	1.58(1.05;2.39)
Model 2	1.00 (ref.)	1.38(1.15;1.64)	1.00(ref.)	1.84(1.51;2.23)	1.00(ref.)	1.54(1.25;1.88)
Model 3	1.00 (ref.)	1.35(1.14;1.62)	1.00(ref.)	1.86(1.53;2.27)	1.00(ref.)	1.62(1.32;2.00)
Model 4	1.00 (ref.)	1.45(1.22;1.74)	1.00(ref.)	2.01(1.65;2.44)	1.00(ref.)	1.60(1.30;1.97)
Model 5	1.00 (ref.)	1.39(1.16;1.67)	1.00(ref.)	1.85(1.51;2.26)	1.00(ref.)	1.60(1.30;1.98)

Data were expressed as OR and 95% CI, using multinomial logistic regression model adjusting for some covariates

^a^ Model 1: Sex + residence + age; Model 2: Model 1 + change in smoking and drinking; Model 3: Model 1 + change in OPAL; Model 4: Model 1 + change in TDEI; Model 5: Model 1 + change in smoking, drinking, OPAL and TDEI. ref: reference;

^b^ Father’s and participant’s occupational position were categorized into high (social classes I–II), medium (social classes III–IV) and low (social class V). Adult education was grouped into high (≥ 12 years formal education), medium (8–11 years formal education), and low (< 8 years formal education)

### The association of life-course socioeconomic changes with BMI trajectories

Table 4 shows the results for the association of life-course socioeconomic changes with BMI trajectories. The risk of progressive overweight among participants with low SEP in childhood but high SEP in adulthood was 1.90 (95% CI: 1.47, 2.46) times higher than those with a stable-low SEP (low SEP in both childhood and adulthood) after adjustment for potential covariates. The stable high life-course SEP was associated with an increased risk of progressive overweight (OR = 1.47, 95% CI: 1.19, 1.84). Similarly, the upward (OR = 1.80, 95% CI: 1.12, 2.90) and stable high (OR = 1.70, 95% CI: 1.13, 2.54) life-course SEP also led to higher risks for progressive obesity. After stratifying by sex, males who were socially upwardly mobile or stable high during the follow-up, had 2.35 and 1.96-fold risks of progressive overweight, and 2.31 and 2.52-fold risks of progressive obesity than those who had a stable-low SEP (Additional file 1: Table S9). Among female participants (Additional file 1: Table S12), stable high SEP in both childhood and adulthood was associated with lower risks of progressive obesity (OR = 0.63, 95% CI: 0.43,0.94) and progressive overweight (OR = 0.53, 95% CI: 0.43,0.66).

**Table 4:** The association of life-course socioeconomic changes with BMI trajectories ^a^

	Progressive overweight vs. normal-stable BMI			
	Stable low	Upward	Downward	Stable high
	OR (95% CI)	OR (95% CI)	OR (95% CI)	OR (95% CI)
Model 1	1.00 (ref.)	1.85(1.44;2.36)	1.10(0.80;1.52)	1.41(1.14;1.73)
Model 2	1.00 (ref.)	1.86(1.45;2.38)	1.11(0.80;1.52)	1.41(1.15;1.74)
Model 3	1.00 (ref.)	1.85(1.44;2.39)	1.08(0.78;1.49)	1.41(1.13;1.75)
Model 4	1.00 (ref.)	1.88(1.47;2.42)	1.17(0.85;1.62)	1.47(1.19;1.82)
Model 5	1.00 (ref.)	1.90(1.47;2.46)	1.13(0.82;1.57)	1.47(1.19;1.84)
	Progressive obesity vs. normal-stable BMI			
	Stable low	Upward	Downward	Stable high
	OR (95% CI)	OR (95% CI)	OR (95% CI)	OR (95% CI)
Model 1	1.00 (ref.)	1.75(1.10;2.79)	1.54(0.89;2.67)	1.70(1.15;2.51)
Model 2	1.00 (ref.)	1.73(1.09;2.76)	1.54(0.89;2.68)	1.65(1.12;2.45)
Model 3	1.00 (ref.)	1.79(1.12;2.88)	1.45(0.83;2.53)	1.65(1.11;2.46)
Model 4	1.00 (ref.)	1.77(1.11;2.83)	1.55(0.89;2.72)	1.80(1.21;2.67)
Model 5	1.00 (ref.)	1.80(1.12;2.90)	1.53(0.87;2.67)	1.70(1.13;2.54)

Data were expressed as OR and 95% CI, using multinomial logistic regression model adjusting for some covariates

^a^ Model 1: Sex + residence + age; Model 2: Model 1 + change in smoking and drinking; Model 3: Model 1 + change in OPAL; Model 4: Model 1 + change in TDEI; Model 5: Model 1 + change in smoking, drinking, OPAL, and TDEI. ref: reference;

Life-course socioeconomic trajectory was computed using information on the father’s and own occupational position, in which each SEP indicator was dichotomized as two levels

### The association of cumulative socioeconomic score with BMI trajectories

In the analysis adjusted for sex, residence, age, change in smoking, drinking, OPAL and TDEI, participants with the highest life-course cumulative SEP score had 2.25 times (95% CI: 1.56, 2.96) and 1.65 times (95% CI: 1.41, 2.06) higher risk of progressive obesity and progressive overweight, respectively during follow-up than those with the lowest life-course cumulative SEP score (Table 5). Among male participants, the highest life-course cumulative SEP score was still associated an increasing risk of progressive obesity and progressive overweight during follow-up (Additional file 1: Table S10). Among the females, in contrast, the highest life-course cumulative SEP score decreased the risks for progressive obesity (OR = 0.20, 95% CI: 0.07, 0.59) and progressive overweight (OR = 0.60, 95% CI: 0.42, 0.85) during follow-up (Additional file 1: Table S13).

**Table 5:** The association of cumulative socioeconomic score with BMI trajectories ^a^

Progressive overweight vs. normal-stable BMI	Cumulative socioeconomic score	
	Lowest	Highest
	OR (95% CI)	OR (95% CI)
Model 1	1.00 (ref.)	1.58(1.32;1.89)
Model 2	1.00 (ref.)	1.59(1.32;1.90)
Model 3	1.00 (ref.)	1.56(1.31;1.83)
Model 4	1.00 (ref.)	1.60(1.39;2.00)
Model 5	1.00 (ref.)	1.65(1.41;2.06)
Progressive obesity vs. normal-stable BMI	Cumulative socioeconomic score	
	Lowest	Highest
	OR (95% CI)	OR (95% CI)
Model 1	1.00 (ref.)	2.19(1.60;3.00)
Model 2	1.00 (ref.)	2.11(1.53;2.89)
Model 3	1.00 (ref.)	2.15(1.56;2.96)
Model 4	1.00 (ref.)	2.34(1.71;3.22)
Model 5	1.00 (ref.)	2.25(1.62;3.10)

Data were expressed as OR and 95% CI, using multinomial logistic regression model adjusting for some covariates

^a^ Model 1: Sex + residence + age; Model 2: Model 1 + change in smoking and drinking; Model 3: Model 1 + change in OPAL; Model 4: Model 1 + change in TDEI; Model 5: Model 1 + change in smoking, drinking, OPAL, and TDEI. ref: reference

Cumulative socioeconomic score (range 0–6) was calculated by summing all SEP indicators, including father’s occupational position, participant’s education and adult occupational position. Each SEP indicator was a 3-level variable with values ranging from 0 (low) to 2 (high)

### Sensitivity analyses

In the study, 121 participants developed diabetes, hypertension, myocardial infarction, stroke, asthma or cancer at the baseline, of whom 14 developed at least two chronic diseases (Additional file 1: Table S14). After excluding these 121 participants, the results indicated that the association of SEP indicators in early life and adult life with BMI trajectories remained similar to the main findings (Additional file 1: Figure S2, Figure S3, Figure S4). Details about the missing covariates were shown in Additional file 1: Table S15. After using multiple imputations to impute missing values of all covariates, we repeated all analyses and observed similar findings (Additional file 1: Figure S5, Figure S6, Figure S7). The results showed that the associations between baseline SEP (father’s occupational position / adult occupational position / adult education) and life-course SEP and BMI trajectories were not mediated by lifestyle behaviors. After adjusting covariates in the BMI trajectory modeling using GMM, the three-class model was identified as the optimal model for the BMI trajectory group (Table S16), and the results remained consistent with the main findings (Figure S8-Figure S10). The relationship between the highest educational attainment and BMI trajectories (Figure S11) was consistent with the main findings. Additionally, no changes were observed in the relationship between adult occupation, educational attainment, and BMI trajectories in the largest possible sample (Table S17 and Table S18).

## Discussion

In the longitudinal survey in China, we explored the relationship between lifetime-specific and life-course SEP and BMI development trajectories adjusting for some covariates. Two indicators of life-course SEP were established, including a measure of SEP changes from childhood to adulthood and a cumulative score of individual SEP through a life time. Our study also identified three patterns of the BMI development trajectories in adulthood among participants using GMM. The associations between life-course SEP and BMI development trajectories differed significantly by sex. In males, experiencing upward and stable high life-course SEP changes and having the highest cumulative SEP score throughout their life were associated with an increased risk of progressive obesity and overweight development. Conversely, among females, these relationships exhibited an inverse pattern.

Our findings showed that the father’s occupational position, which indicated the SEP of participants in childhood, was associated with the increasing risk of progressive obesity in adulthood among males. However, no such association was found among females. Similar to our results, a China cohort study reported a positive association between childhood SEP, measured by parental possession, and waist circumference in adult males [32]. Higher parental SEP in childhood had significant association with increased BMI in young Filipino males (β = 2.04) [33]. Studies also showed that socioeconomic position in early life and perhaps even in earlier generations have significant influence on adult obesity development [34–36]. There are several explanations for how exposure to low SEP in early life influences health later in life. First, the impact of SEP-related factors on health varied with different developmental stages, and the effects were greatest in specific stages (e.g. in utero, early childhood, adolescence). Ziol-Guest et al. confirmed that in the prenatal period and the first year of life rather than in other periods of childhood, the family income had significant effect on adult BMI [37]. Second, the effect of poor SEP in early life on adult health changed with the intensity and duration of exposure to socioeconomic disadvantage. Evidences suggested that a longer duration of exposure to early childhood poverty would be associated with accelerated BMI growth trajectories in the future [38].

In our study, a significant positive association was found between adulthood SEP and adult overweight/obesity in males, consistent with those in low-middle income countries [39, 40]. Among females, however, the adult occupation was inversely associated with the obesity. Although the negative correlation between education, occupation, and obesity was commonly observed among females in high-income countries [41–43], a recent review indicated a similar relationship among females in middle-income countries [8]. It is plausible that females occupying higher positions in the workforce experience heightened pressure to maintain slim and exert more control over their weight compared to their peers in lower occupational positions [44]. The SEP and obesity gradient is changing in the opposite direction in low and middle-income countries, resembling that of high-income countries, especially among females [45].

Although the SEP at the baseline was predictive of adulthood obesity, it only reflected the associations between adult overweight and obesity and SEP at specific time points, ignoring the social mobility of this sample. Thus, the life-course socioeconomic changes and cumulative score were established to reflect the intergenerational mobility of socioeconomic position between parents and children from a life-course perspective [46]. Our results showed that males with upward and stable high SEP, were more likely to suffer from long-term obesity compared with individuals remaining in a low SEP class, consistent with previous studies in low-middle income countries [47, 48]. According to a cross-sectional survey in five middle-income countries, males with stable high educational attainment during the life-course was associated with the increased risks of overweight/obesity [47]. In our study, however, the risk of obesity in females decreased with stable high socioeconomic position from childhood to adulthood, relative to the group with stable low socioeconomic position. Sinead et al. conducted a study in Denmark which revealed that females who experienced a decline in SEP, from adolescence to adulthood, had a higher likelihood of developing overweight or obesity compared to those who maintained a high SEP [42]. Studies suggested that socioeconomic exposures may accumulate during the life-course and contribute to poor health outcomes [49, 50]. Based on an accumulation of risk model, a cumulative socioeconomic score from early life to adulthood was positively associated with progressive obesity trajectory in males in our study, but not in females. The study findings indicate that both individual SEP mobility and the cumulative life-course SEP have a gender-specific impact on the risk of obesity. Individuals residing in countries undergoing socioeconomic shifts are profoundly influenced by social and environmental factors, including a sedentary lifestyle and the accessibility of nutritious food. Moreover, additional research is necessary to formulate public health strategies that mitigate the influence of socioeconomic shifts in early life on adult health. Special attention should be given to males experiencing upward socioeconomic change. The parents can play an active role by focusing on promoting healthy dietary habits, encouraging regular exercise, and facilitating BMI self-testing.

Previous studies have indicated the effect of SEP on weight status mediated by health-related behaviors, such as leisure-time physical activity [51] and fruit and vegetables intake [52]. Unfortunately, our study did not find any significant mediation effects of health-related behaviors on the association between SEP and obesity. These findings highlight the complexity of the relationship between SEP and BMI, and more longitudinal studies are required to gain a more meaningful insight.

Based on a 20-year follow-up cohort in China, we can clearly confirm the association between life-course socioeconomic position and the risk of overweight/obesity. Additionally, we established the BMI development trajectories using GMM, which was a more stable and reliable indicator to reflect the long-term change in BMI. The two measures of life-course SEP (life-course socioeconomic trajectories and a cumulative SEP index) were also adopted to explore their origins of SEP disparities in early life and the long-term effect of SEP on obesity. In this study, however, some limitations should be noted in the explanation of the results. Firstly, the socioeconomic level and lifestyle were mainly self-reported by participants, thus the possibility of information bias was not ruled out. Second, because of a lack of data on parents’ income and education and other related SEP indicators that represent the SEP in the early life of participants, the life course SEP variable was constructed only using paternal occupation and the participant’s occupation. Future studies with more reliable SEP indicators in early life are preferred. Third, we excluded those with major chronic diseases at the baseline to minimize the possible reverse causation from these diseases. However, the possibility of reverse causation and residual confounding may still exist in our study due to many other unmeasured diseases. Fourth, our results may be subjected to other potential confounders that were not observed in the study. Fifth, although CHNS is a nationally representative sample, 35,398 participants were excluded and only 3138 eligible adults were included. The reason for this was that in our analyses less than 50% of the individuals could match the parent-child relationship. In addition, tracking the careers of two generations requiring a longer time span, will lead to more missing data. However, the results of sensitivity analysis considering only the completeness of SEP, such as occupation/education in adulthood, for a larger sample, showed that the associations were robust.

## Conclusion

Our study found a significant positive relationship between both childhood and adulthood SEP and adult overweight/obesity among males and the inverse relationship in females. The upward and stable high life-course SEP changes, and the highest life-course cumulative SEP score led to higher risks for progressive obesity and overweight among males, but the relationship among females was the opposite. Taking into account the gender disparities in obesity related to socioeconomic position, special emphasis should be placed on males experiencing upward and stable socioeconomic change.

### Electronic supplementary material

Below is the link to the electronic supplementary material.

Supplementary Material 1

## Data Availability

China Health and Nutrition Survey data are available in a public, open access website (https://www.cpc.unc.edu/projects/china/data/datasets.) The data that support the findings of this study are available from the corresponding author Leilei Pei.

## Abbreviations

AR: Autoregressive structure
AIC: Akaike information criterion
BIC: Bayesian information criterions
BMI: Body mass index
CHNS: China Health and Nutrition Survey
DAG: Directed acyclic graph
GMM: The growth mixture model
IR: Interquartile range
SEP: Socioeconomic position
VIF: Variance inflation factor
