# Supplementary material for: Life-course social disparities in body mass index trajectories across adulthood: cohort study evidence from China health and nutrition survey
Source: BMC Public Health. 2023 Oct 9;23:1955. doi: 10.1186/s12889-023-16881-4 (PMC10563291; doi:10.1186/s12889-023-16881-4)
Supplement: Supplementary file 1 — Supplementary Material 1 [file 12889_2023_16881_MOESM1_ESM.pdf]

Additional file 1

Text: Growth mixture modelling

We used Mplus (version 8.3) to develop a single growth mixture model that identified distinct groups of individuals who had similar BMI trajectories during the 20-year follow-up.

BMI was assessed at 10 waves used in the present study. Mplus software requires the data to be in wide format, with a separate column for BMI data at each wave. As described in the methods, we excluded individuals who did not have two or more serial BMI measurements. **Additional file 1—Table S3** provides a detailed description of the waves and anthropometric data used for analysis. The age scale was centered at the mean of wave_2000 and wave_2004 (30.5 years) to aid numerical stability. The growth mixture model specifications were determined through a series of steps, aiming to improve the Bayesian Information Criterion (BIC) when retaining theoretical plausibility, but it ignored entropy statistics because they do not measure model fit (van de Schoot et al., 2017). For 1-7 class solutions, we fitted a series of mixture models in which the longitudinal BMI response was described using each of the following age functions: linear (Model 1), quadratic (Model 2), cubic polynomial (Model 3) and freely estimated polynomials (Model 4). The quadratic polynomial function provided the best fit for the data (**Additional file 1—Table S4**). Using the quadratic polynomial models, we next tried to relax some of the main default constraints implemented by Mplus. Because the variances of BMI in each wave were different, we assumed heteroscedasticity within BMI and set the BMI

residual variance (i.e. error) to be different across waves. Allowing the residual variances/errors to differ across the classes improved model fit BIC by between 23 and 151 (Model 5 vs. Model 2). We then attempted to extend Model 2 to include a within-class autocorrelation structure for the residual variances/errors. The final model included the class regression of the BMI on BMI $_{t-1}$ to build the autoregressive correlation model (AR (1)). This improved model fit by between 153 and 687 (Model 6 vs. Model 2). Though improved model fit by between 41 and 359 (Model 4 vs. Model 2), the model fitting of the unconstrained model (model 4) was not better than that of final model (Model 6). In summary, the best-fitting model covered a quadratic polynomial function of age, within-class heteroscedastic errors, and a first-order autoregressive structure (AR1) to model auto-correlation. Then the model including a quadratic polynomial function of age, within-class heteroscedastic errors, and a first-order autoregressive structure (AR1) was run for 1-7 class solutions. In the modeling process, we included sex as covariate to adjust the pattern difference between sexes. To avoid convergence at local minima, we performed 1000 random starts (for 20 iterations), of which the best 200 models (according to log-likelihood) were run to completion (STARTS = 2000 200; STITERATIONS = 50). The number of trajectories was chosen based on better goodness of fit ((i.e. lower Akaike Information Criteria (AIC) and Bayesian Information Criterion (BIC)), internal reliability (mean posterior probability > 0.7 for each latent class, reflecting an acceptable uncertainty of posterior classification), clinical plausibility and interpretability. As shown in **Additional file 1—Table S5**, the reduction in BIC between model k and model k-1 solutions attenuated exponentially as the number of classes increased. Finally, we found that the reduction in BIC between the 3-class solution and the 4-class solution was greatest. The

entropy was slightly higher in the 3-class solution, while the average posterior probabilities of class membership were always greater than the proposed cut-off of 0.70 in the 3-class solution but not in the 4-class solution. All classes in the 3-class solution comprised a reasonable number of participants, with the lowest proportion of individuals being 7.00% (class 1, progressive obesity group). However, the smallest proportion in the 4-class solution was 5.00%. We therefore selected the 3-class solution as our final model.

Table S1. Social class based on self-reported primary occupation

Social class	Primary occupation
Class I	1. Senior professional/technical worker
Class II	1. Junior professional/technical worker
	2. Administrator/executive/manager
	3. Army officer, police officer
Class III (skilled manual)	1. Skilled worker
Class III (skilled non-manual)	2. Office staff
	3. Soldier, policeman
	4. Athlete, actor, musician
Class IV (semi-skilled)	1. Driver
	2. Service worker
Class V (unskilled)	1. Farmer, fisherman, hunter
	2. Non-skilled worker

Table S2-1. Definition of cumulative socioeconomic score during the life-course

Indicators	Min	Max	Range
Father’s occupational position	0	2	2
Adult occupational position	0	2	2
Adult education	0	2	2
Cumulative socioeconomic scores	0	6	6
	(The lowest)	(The highest)	

The cumulative SEP score during the life-course was derived using information on paternal occupation and participant’s education and adult occupation. According to the classification criteria of socioeconomic classification scheme in Additional file 1—Table S1, father’s and participants’ occupation were categorized into high (social classes I–II), medium (social classes III–IV) and low (social class V). The three individual SEP indicators were each coded 0(low)-2(high), and all indicators were summed, ranging from 0 to 6, with higher values corresponding to greater life-course advantage.

Table S2-2. Definition of life-course socioeconomic trajectories

Father’s occupational position	Adult occupational position	
	Low	High
Low	stable low	upward
High	downward	stable high

The life-course socioeconomic trajectories from childhood to adulthood were computed using information on the father’s occupation and adult occupation, both of which were dichotomized as high (social class I-IV) and low (social class V) based on Additional file 1—Table S1.

Table S3. Description of the longitudinal anthropometric data during the follow-up

Visit	Age (years)						BMI (kg/m^2)			
	25th centile	50th centile	75th centile	Min	Max	Range	N	25th centile	50th centile	75th centile
Wave_1989	19.00	22.00	26.00	18.00	47.00	29.00	721	19.38	20.58	22.22
Wave_1991	20.00	22.00	27.00	18.00	67.00	49.00	1313	19.51	21.08	22.49
Wave_1993	21.00	24.00	28.00	18.00	69.00	51.00	1343	19.83	21.36	22.88
Wave_1997	23.00	27.00	31.00	18.00	71.00	53.00	1178	19.93	21.81	23.16
Wave_2000	25.00	29.00	33.00	18.00	70.00	52.00	1194	19.88	22.89	24.43
Wave_2004	27.00	32.00	36.00	18.00	71.00	53.00	1113	21.27	23.18	25.31
Wave_2006	28.00	34.00	38.00	18.00	73.00	55.00	1070	21.64	24.08	25.23
Wave_2009	30.00	36.00	41.00	18.00	75.00	57.00	1221	21.22	24.20	26.04
Wave_2011	32.00	38.00	43.00	18.00	77.00	59.00	1176	22.18	25.16	26.25
Wave_2015	36.00	42.00	47.00	22.00	81.00	59.00	1111	21.08	24.94	27.57

Table S4. Comparison of the BIC between mixture models (1-7 classes) with different specifications

	**Model 1**	**Model 2**		**Model 3**		**Model 4**		**Model 5**		**Model 6**	
	Linear	Quadratic polynomial		Cubic polynomial		GMM _free		Model 2 +Residual variances (Of BMI measures) allowed to differ across classes)		Model 5 + inclusion autoregression structure [a]	
	BIC	BIC	ΔBIC from model 1	BIC	ΔBIC from model 2	BIC	ΔBIC from model 2	BIC	ΔBIC from model 2	BIC	ΔBIC from model 2
Class											
1	49991	49794	-197	49968	174	50219	425	49643	-151	49510	-284
2	49734	49440	-293	49629	189	49399	-41	49330	-110	49287	-153
3	49979	49204	-775	49523	319	49040	-164	49066	-138	48906	-298
4	49547	49148	-398	49164	16	48875	-273	49044	-104	48656	-492
5	49501	49123	-379	49039	-84	48764	-359	49100	-23	48482	-641
6	49465	49079	-386	49079	0	-	-	48997	-82	48415	-664
7	49454	49068	-386	48970	-98	-	-	48922	-146	48381	-687

[a] autoregression of adjacent BMI measurements

Table S5. Summary of final mixture models (1-7 classes)

Classes	1	2	3	4	5	6	7
AIC	49400	49136	48839	48573	48383	48300	48250
BIC	49510	49287	48906	48656	48482	48415	48381
BIC difference	--	-223	-381	-266	-190	-82	-49
Entropy	--	0.62	0.54	0.46	0.50	0.53	0.51
Posterior probability [mean]							
Class-1	--	0.90	0.82	0.81	0.62	0.70	0.68
Class-2	--	0.84	0.78	0.63	0.85	0.59	0.48
Class-3	--		0.75	0.69	0.68	0.90	0.89
Class-4	--			0.67	0.63	0.67	0.60
Class-5	--				0.72	0.62	0.69
Class-6	--					0.71	0.59
Class-7	--						0.64
Class membership [N (%)]							
Class-1	--	528(0.17)	223(0.07)	180(0.06)	44(0.01)	30(0.01)	30(0.01)
Class-2		2610(0.83)	988(0.31)	678(0.22)	385(0.12)	96(0.03)	97(0.03)
Class-3			1927(0.61)	1078(0.34)	670(0.21)	315(0.10)	164(0.05)
Class-4				1202(0.38)	847(0.27)	723(0.23)	438(0.14)
Class-5					1192(0.38)	778(0.25)	732(0.23)
Class-6						1196(0.38)	742(0.24)
Class-7							935(0.30)

Table S6. A GRoLTS-Checklist for this study

Checklist Item	Reported? "Yes/No"	If "Yes", Where?	If "No", Why?
1. Is the metric of time used in the statistical model reported?	Yes, specified as year.	Throughout the manuscript	
2. Is information presented about the mean and variance of time within a wave?	Yes.	**Additional file 1—Table S3**	.
3a. Is the missing data mechanism reported?	Yes.	Statistical Analysis Segment of the Methods Section	
3b. Is a description provided of what variables are related to attrition/missing data?	Yes.		We compare individuals who have dropped out to individuals who completed the study in Additional file 1—Table S7
3c. Is a description provided of how missing data in the analyses were dealt with?	Yes	Statistical Analysis Segment of the Methods Section	
4. Is information about the distribution of the observed variables included?	Yes	Additional file 1—Table S3	
5. Is the software mentioned?	Yes	Statistical Analysis Segment of the Methods Section	
6a. Are alternative specifications of within-class heterogeneity considered (e.g., LGCA vs. LGMM) and clearly documented? If not, was sufficient justification provided as to eliminate certain specifications from consideration?	Yes	Statistical Analysis Segment of the Methods Section; Results Section	

6b. Are alternative specifications of the between-class differences in variance–covariance matrix structure considered and clearly documented? If not, was sufficient justification provided as to eliminate certain specifications from consideration?	Yes	Statistical Analysis Segment of the Methods Section, Results Section	
6. Are alternative shape/functional forms of the trajectories described?	Yes	Statistical Analysis Segment of the Methods Section; Results Section	
8. If covariates have been used, can analyses still be replicated?	Yes.	Results section.	
9. Is information reported about the number of random start values and final iterations included?	Yes	Additional file 1—Text.	
10. Are the model comparison (and selection) tools described from a statistical perspective?	Yes.	Statistical Analysis Segment of the Methods Section.	
11. Are the total number of fitted models reported, including a one-class solution?	Yes	Statistical Analysis Segment of the Methods Section; Additional file 1—Table S4.- Additional file 1—Table S5.	
12. Are the number of cases per class reported for each model (absolute sample size, or proportion)?	Yes, proportions	Results Section	
13. If classification of cases in a trajectory is the goal, is entropy reported?	No		Cases were not classified into trajectories

14a. Is a plot included with the estimated mean trajectories of the final solution?	Yes	Figure 2.	
14b. Are plots included with the estimated mean trajectories for each model?	No.		
14c. Is a plot included of the combination of estimated means of the final model and the observed individual trajectories split out for each latent?	No		As attrition was non-monotonic, Plots would be difficult to interpret. Adding that we did not assign cases to classes, we determined there would be minimal marginal gain.
15. Are characteristics of the final class solution numerically described (i.e., means, SD/SE, n, CI, etc.)?	Yes.	Tables.	
16. Are the syntax files available (either in the Additional file, supplementary materials, or from the authors)?	Yes	supplementary materials	

Table S7. Characteristics of participants included and excluded in the analysis [a]

Characteristics	Included sample	Excluded sample
**Participants (n)**	3138	27528
**Age (years)**	23.0 (8.0)	36.4(14.0)
**Female (%)**	909(29.0)	15380(44.1)
**Rural (%)**	2070(66.0)	16244(59.0)
**BMI**	20.76(3.1)	22.41(4.1)
**Adult occupational position**		
Low	1033(32.9)	9348(34.0)
Medium	1331(42.4)	6516(23.7)
High	774(24.7)	5496(20.0)
**Adult education**		
Low	734(23.4)	3727(13.5)
Medium	1398(44.6)	13195(47.9)
High	1006(32.1)	9657(35.1)
Initial health-related behaviors at baseline		
**Cigarette smoker**	847（26.4）	5922(21.5)
**Alcohol drinker**	852（26.6）	6743(24.5)
**OPAL**		
Light OPAL	841(27.2)	10753(39.1)
Moderate OPAL	1086(34.6)	5272(19.2)
Heavy OPAL	1147(36.5)	6709(24.4)
**TDEI (kcal)**	2261.0(900.2)	2200.4(960.8)

[a] Data were expressed as numbers (percentages). Non-normally distributed data like baseline age was reported as median (IQR).

BMI: body mass index; OPAL: occupational physical activity level; TDEI: total daily energy intake.

Table S8. The association of socioeconomic position in early life and adult life with BMI trajectories [a, b]: Males

	Progressive overweight vs. normal-stable BMI					
	Father's occupational position		Adult occupational position		Adult education	
	Low	High	Low	High	Low	High
	OR (95% CI)	OR (95% CI)	OR (95% CI)	OR (95% CI)	OR (95% CI)	OR (95% CI)
Model 1	1.00(ref.)	1.34(1.07;1.68)	1.00(ref.)	2.36(2.07;2.69)	1.00(ref.)	1.55(1.37;1.76)
Model 2	1.00(ref.)	1.34(1.07;1.68)	1.00(ref.)	2.38(2.08;2.71)	1.00(ref.)	1.56(1.37;1.77)
Model 3	1.00(ref.)	1.35(1.07;1.70)	1.00(ref.)	2.43(2.12;2.78)	1.00(ref.)	1.60(1.40;1.81)
Model 4	1.00(ref.)	1.37(1.09;1.72)	1.00(ref.)	2.40(2.10;2.74)	1.00(ref.)	1.55(1.37;1.76)
Model 5	1.00(ref.)	1.38(1.10;1.75)	1.00(ref.)	2.50(2.18;2.86)	1.00(ref.)	1.60(1.41;1.83)
	Progressive obesity vs. normal-stable BMI					
	Father's occupational position		Adult occupational position		Adult education	
	Low	High	Low	High	Low	High
	OR (95% CI)	OR (95% CI)	OR (95% CI)	OR (95% CI)	OR (95% CI)	OR (95% CI)
Model 1	1.00(ref.)	1.92(1.29;2.86)	1.00(ref.)	3.67(2.92;4.60)	1.00(ref.)	1.84(1.45;2.33)
Model 2	1.00(ref.)	1.87(1.26;2.80)	1.00(ref.)	3.53(2.80;4.44)	1.00(ref.)	1.78(1.40;2.26)
Model 3	1.00(ref.)	1.81(1.21;2.70)	1.00(ref.)	3.58(2.83;4.53)	1.00(ref.)	1.87(1.47;2.38)
Model 4	1.00(ref.)	1.98(1.33;2.96)	1.00(ref.)	3.72(2.96;4.68)	1.00(ref.)	1.84(1.45;2.33)
Model 5	1.00(ref.)	1.85(1.24;2.78)	1.00(ref.)	3.56(2.80;4.52)	1.00(ref.)	1.84(1.44;2.35)

[a] Model 1: Gender +residence +age; Model 2: Model 1+ change in smoking and drinking; Model 3: Model 1+ change in OPAL; Model 4: Model 1+ change in TDEI; Model 5: Model 1 + change in smoking, drinking, OPAL, and TDEI. ref: reference; OPAL: occupational physical activity level; TDEI: total daily energy intake.

[b] Father's and participants' occupation were categorized into high (social classes I–II), medium (social classes III–IV) and low (social class V). Adult education was grouped into high (≥12 years formal education), medium (8-11 years formal education), and low (<8 years formal

education).

Table S9. The association of life-course socioeconomic changes with BMI trajectories [a, b]: Males

	Progressive overweight vs. normal-stable BMI			
	Stable low	Upward	Downward	Stable high
	OR (95% CI)	OR (95% CI)	OR (95% CI)	OR (95% CI)
Model 1	1.00(ref.)	2.35(2.05;2.71)	1.16(0.95;1.40)	1.96(1.74;2.21)
Model 2	1.00(ref.)	2.37(2.06;2.73)	1.16(0.95;1.40)	1.97(1.74;2.23)
Model 3	1.00(ref.)	2.41(2.09;2.79)	1.16(0.95;1.41)	2.03(1.79;2.31)
Model 4	1.00(ref.)	2.40(2.08;2.76)	1.21(0.98;1.46)	2.03(1.79;2.29)
Model 5	1.00(ref.)	2.48(2.14;2.87)	1.20(0.98;1.46)	2.11(1.86;2.40)
	Progressive obesity vs. normal-stable BMI			
	Stable low	Upward	Downward	Stable high
	OR (95% CI)	OR (95% CI)	OR (95% CI)	OR (95% CI)
Model 1	1.00(ref.)	2.21(1.68;2.91)	1.42(0.99;2.03)	2.56(2.03;3.23)
Model 2	1.00(ref.)	2.19(1.66;2.88)	1.43(0.99;2.04)	2.48(1.96;3.13)
Model 3	1.00(ref.)	2.26(1.72;2.99)	1.26(0.87;1.82)	2.46(1.94;3.12)
Model 4	1.00(ref.)	2.29(1.74;3.01)	1.50(0.73;2.14)	2.67(2.11;3.38)
Model 5	1.00(ref.)	2.31(1.75;3.06)	1.32(0.91;1.95)	2.52(1.98;3.21)

[a] Model 1: Gender +residence +age; Model 2: Model 1+ change in smoking and drinking; Model 3: Model 1+ change in OPAL; Model 4: Model 1+ change in TDEI; Model 5: Model 1 + change in smoking, drinking, OPAL, and TDEI. ref: reference; OPAL: occupational physical activity level; TDEI: total daily energy intake.

[b] Life-course socioeconomic trajectory was computed using information on the father's occupation and adult occupation, both of which were dichotomized as high (social class I-IV) and low (social class V) based on Additional file 1—Table S1.

Table S10. The association of cumulative socioeconomic score with BMI trajectories [a, b]: Males

Progressive overweight vs. normal-stable BMI	Lowest	Highest
	OR (95% CI)	OR (95% CI)
Model 1	1.00(ref.)	2.58(2.07;3.21)
Model 2	1.00(ref.)	2.60(2.08;3.24)
Model 3	1.00(ref.)	2.77(2.21;3.47)
Model 4	1.00(ref.)	2.89(2.37;3.69)
Model 5	1.00(ref.)	2.90(2.31;3.65)
Progressive obesity vs. normal-stable BMI	Lowest	Highest
	OR (95% CI)	OR (95% CI)
Model 1	1.00(ref.)	3.76(2.62;5.39)
Model 2	1.00(ref.)	3.67(2.56;5.28)
Model 3	1.00(ref.)	3.64(2.53;5.25)
Model 4	1.00(ref.)	3.79(2.63;5.35)
Model 5	1.00(ref.)	3.92(2.70;5.68)

[a] Cumulative socioeconomic score (range 0–6) is calculated by summing all SEP indicators, including father's occupational position, participant's education and adult occupational position. Each SEP indicator was a 3-level variable with values ranging from 0 (low) to 2 (high).

[b] Model 1: Gender +residence +age; Model 2: Model 1+ change in smoking and drinking; Model 3: Model 1+ change in OPAL; Model 4: Model 1+ change in TDEI; Model 5: Model 1 + change in smoking, drinking, OPAL, and TDEI. ref: reference; OPAL: occupational physical activity level; TDEI: total daily energy intake.

Table S11. The association of socioeconomic position in early life and adult life with BMI trajectories [a,b]: Females

	Father's occupational position		Adult occupational position		Adult education	
	**Progressive overweight vs. normal-stable BMI**					
	Lowest	Highest	Lowest	Highest	Lowest	Highest
	OR (95% CI)	OR (95% CI)	OR (95% CI)	OR (95% CI)	OR (95% CI)	OR (95% CI)
Model 1	1.00(ref.)	0.67(0.45;1.00)	1.00(ref.)	0.57(0.36;0.89)	1.00(ref.)	0.64(0.41;1.00)
Model 2	1.00(ref.)	0.68(0.46;1.02)	1.00(ref.)	0.60(0.38;0.94)	1.00(ref.)	0.65(0.42;1.02)
Model 3	1.00(ref.)	0.65(0.43;0.97)	1.00(ref.)	0.56(0.35;0.88)	1.00(ref.)	0.63(0.40;0.98)
Model 4	1.00(ref.)	0.69(0.47;1.04)	1.00(ref.)	0.58(0.37;0.91)	1.00(ref.)	0.66(0.42;1.03)
Model 5	1.00(ref.)	0.68(0.45;1.03)	1.00(ref.)	0.59(0.37;0.94)	1.00(ref.)	0.66(0.41;1.04)
	**Progressive obesity vs. normal-stable BMI**					
	Father's occupational position		Adult occupational position		Adult education	
	Lowest	Highest	Lowest	Highest	Lowest	Highest
	OR (95% CI)	OR (95% CI)	OR (95% CI)	OR (95% CI)	OR (95% CI)	OR (95% CI)
Model 1	1.00(ref.)	0.53(0.24;1.13)	1.00(ref.)	0.28(0.10;0.72)	1.00(ref.)	1.16(0.50;2.65)
Model 2	1.00(ref.)	0.53(0.24;1.14)	1.00(ref.)	0.27(0.10;0.70)	1.00(ref.)	1.11(0.48;2.55)
Model 3	1.00(ref.)	0.54(0.25;1.18)	1.00(ref.)	0.27(0.11;0.72)	1.00(ref.)	1.21(0.52;2.78)
Model 4	1.00(ref.)	0.58(0.27;1.25)	1.00(ref.)	0.30(0.12;0.78)	1.00(ref.)	1.18(0.52;2.70)
Model 5	1.00(ref.)	0.60(0.27;1.31)	1.00(ref.)	0.28(0.11;0.75)	1.00(ref.)	1.20(0.52;2.76)

[a] Model 1: Gender +residence +age; Model 2: Model 1+ change in smoking and drinking; Model 3: Model 1+ change in OPAL; Model 4: Model 1+ change in TDEI; Model 5: Model 1 + change in smoking, drinking, OPAL, and TDEI. ref: reference; OPAL: occupational physical activity level; TDEI: total daily energy intake.

[b] Father's and participants' occupation were categorized into high (social classes I–II), medium (social classes III–IV) and low (social class V). Adult education was grouped into high (≥12 years formal education), medium (8-11 years formal education), and low (<8 years formal

education).

Table S12. The association of life-course socioeconomic changes with BMI trajectories [a,b]: Females

	Stable low	Upward	Downward	Stable high
	Progressive overweight vs. normal-stable BMI			
	Stable low	Upward	Downward	Stable high
	OR (95% CI)	OR (95% CI)	OR (95% CI)	OR (95% CI)
Model 1	1.00(ref.)	0.87(0.66;1.15)	0.82(0.62;1.08)	0.54(0.44;0.66)
Model 2	1.00(ref.)	0.88(0.66;1.17)	0.82(0.62;1.07)	0.55(0.45;0.68)
Model 3	1.00(ref.)	0.86(0.65;1.45)	0.76(0.57;1.01)	0.50(0.40;0.61)
Model 4	1.00(ref.)	0.91(0.68;1.20)	0.88(0.67;1.17)	0.57(0.46;0.70)
Model 5	1.00(ref.)	0.89(0.67;1.19)	0.80(0.60;1.07)	0.53(0.43;0.66)
	Progressive obesity vs. normal-stable BMI			
	Stable low	Upward	Downward	Stable high
	OR (95% CI)	OR (95% CI)	OR (95% CI)	OR (95% CI)
Model 1	1.00(ref.)	1.10(0.68;1.76)	1.40(0.91;2.16)	0.58(0.39;0.84)
Model 2	1.00(ref.)	1.16(0.72;1.88)	1.48(0.95;2.29)	0.57(0.39;0.83)
Model 3	1.00(ref.)	1.14(0.71;1.83)	1.45(0.94;2.24)	0.59(0.41;0.87)
Model 4	1.00(ref.)	1.00(0.61;1.63)	1.49(0.96;2.30)	0.63(0.43;0.91)
Model 5	1.00(ref.)	1.10(0.67;1.82)	1.65(1.06;2.57)	0.63(0.43;0.94)

[a] Model 1: Gender +residence +age; Model 2: Model 1+ change in smoking and drinking; Model 3: Model 1+ change in OPAL; Model 4: Model 1+ change in TDEI; Model 5: Model 1 + change in smoking, drinking, OPAL, and TDEI. ref: reference; OPAL: occupational physical activity level; TDEI: total daily energy intake.

[b] Life-course socioeconomic trajectory was computed using information on the father's occupation and adult occupation, both of which were dichotomized as high (social class I-IV) and low (social class V) based on Additional file 1—Table S1.

Table S13. The association of cumulative socioeconomic score with BMI trajectories [a,b]: Females

Progressive overweight vs. normal-stable BMI	Lowest	Highest
	OR (95% CI)	OR (95% CI)
Model 1	1.00(ref.)	0.56(0.40;0.78)
Model 2	1.00(ref.)	0.58(0.41;0.82)
Model 3	1.00(ref.)	0.54(0.38;0.76)
Model 4	1.00(ref.)	0.59(0.43;0.84)
Model 5	1.00(ref.)	0.60(0.42;0.85)
Progressive obesity vs. normal-stable BMI	Lowest	Highest
	OR (95% CI)	OR (95% CI)
Model 1	1.00(ref.)	0.18(0.06;0.55)
Model 2	1.00(ref.)	0.18(0.06;0.53)
Model 3	1.00(ref.)	0.19(0.06;0.57)
Model 4	1.00(ref.)	0.20(0.07;0.59)
Model 5	1.00(ref.)	0.20(0.07;059)

[a] Cumulative socioeconomic score (range 0–6) is calculated by summing all SEP indicators, including father's occupational position, participant's education and adult occupational position. Each SEP indicator was a 3-level variable with values ranging from 0 (low) to 2 (high).

[b] Model 1: Gender +residence +age; Model 2: Model 1+ change in smoking and drinking; Model 3: Model 1+ change in OPAL; Model 4: Model 1+ change in TDEI; Model 5: Model 1 + change in smoking, drinking, OPAL, and TDEI. ref: reference; OPAL: occupational physical activity level; TDEI: total daily energy intake.

Table S14. Details of chronic diseases of participants at the baseline (n=121)

Chronic Disease	Number (%)
HYPERTENSION	79(2.5)
DIABATES	29(0.9)
STROKE	7(0.2)
MYOCARDIAL INFARCTION	5(0.2)
ASTHMA	9(0.3)
CANCER	6(0.2)

Note: There were 14 participants with two or more chronic diseases.

Table S15. Details of missing covariates in the sample

Variable	Missing ratio (%)
Smoking	1(0.1)
Drinking	3(0.1)
OPAL	80(2.5)
TDEI	77(2.5)

OPAL: occupational physical activity level; TDEI: total daily energy intake.

Table S16. The comparison of the mixture models (GMM & GMM adjusted for the covariates)

	GMM (Model 6 [a])	GMM (Model 6 adjusted for the covariates)
AIC	48839	48780
BIC	48906	48932
Entropy	0.540	0.545
Probability		
Class-1	0.82	0.82
Class-2	0.78	0.79
Class-3	0.75	0.75
Membership [N (%)]		
Class-1	223(0.07)	223(0.07)
Class-2	988(0.31)	992(0.31)
Class-3	1927(0.61)	1923(0.61)

[a] The model with quadratic polynomial function of age + Residual variances+ inclusion autoregression structure (AR1)

Table S17. The association between adult SEP and BMI trajectories in a large sample (Males)

	**Analysis sample（N=3,138）**				**Sample with complete adult socioeconomic positions(N=18.806)**			
	Progressive overweight vs. normal-stable BMI				Progressive overweight vs. normal-stable BMI			
	Adult occupational position		Adult education		Adult occupational position		Adult education	
	Lowest	Highest	Lowest	Highest	Lowest	Highest	Lowest	Highest
	OR (95% CI)	OR (95% CI)	OR (95% CI)	OR (95% CI)	OR (95% CI)	OR (95% CI)	OR (95% CI)	OR (95% CI)
Model 1	1.00(ref.)	2.36(2.07;2.69)	1.00(ref.)	1.55(1.37;1.76)	1.00(ref.)	2.61(2.38;2.86)	1.00(ref.)	3.26(2.87;3.70)
Model 2	1.00(ref.)	2.38(2.08;2.71)	1.00(ref.)	1.56(1.37;1.77)	1.00(ref.)	2.56(2.33;2.80)	1.00(ref.)	3.09(2.72;3.52)
Model 3	1.00(ref.)	2.43(2.12;2.78)	1.00(ref.)	1.60(1.40;1.81)	1.00(ref.)	2.62(2.39;2.88)	1.00(ref.)	3.23(2.83;3.68)
Model 4	1.00(ref.)	2.40(2.10;2.74)	1.00(ref.)	1.55(1.37;1.76)	1.00(ref.)	2.59(2.36;2.84)	1.00(ref.)	3.18(2.80;3.62)
Model 5	1.00(ref.)	2.50(2.18;2.86)	1.00(ref.)	1.60(1.41;1.83)	1.00(ref.)	2.56(2.33;2.81)	1.00(ref.)	3.02(2.64;3.44)
	Progressive obesity vs. normal-stable BMI				Progressive obesity vs. normal-stable BMI			
	Adult occupational position		Adult education		Adult occupational position		Adult education	
	Lowest	Highest	Lowest	Highest	Lowest	Highest	Lowest	Highest
	OR (95% CI)	OR (95% CI)	OR (95% CI)	OR (95% CI)	OR (95% CI)	OR (95% CI)	OR (95% CI)	OR (95% CI)
Model 1	1.00(ref.)	3.67(2.92;4.60)	1.00(ref.)	1.84(1.45;2.33)	1.00(ref.)	6.12(5.34;7.01)	1.00(ref.)	7.62(6.40;9.07)
Model 2	1.00(ref.)	3.53(2.80;4.44)	1.00(ref.)	1.78(1.40;2.26)	1.00(ref.)	5.86(5.11;6.72)	1.00(ref.)	6.72(5.64;8.02)
Model 3	1.00(ref.)	3.58(2.83;4.53)	1.00(ref.)	1.87(1.47;2.38)	1.00(ref.)	6.28(5.47;7.21)	1.00(ref.)	7.49(6.26;8.96)
Model 4	1.00(ref.)	3.72(2.96;4.68)	1.00(ref.)	1.84(1.45;2.33)	1.00(ref.)	6.10(5.32;7.00)	1.00(ref.)	7.49(6.28;8.92)
Model 5	1.00(ref.)	3.56(2.80;4.52)	1.00(ref.)	1.84(1.44;2.35)	1.00(ref.)	5.99(5.20;6.90)	1.00(ref.)	6.62(5.52;7.95)

[a] Model 1: Gender +residence +age; Model 2: Model 1+ change in smoking and drinking; Model 3: Model 1+ change in OPAL; Model 4: Model 1+ change in TDEI; Model 5: Model 1 + change in smoking, drinking, OPAL, and TDEI. ref: reference; OPAL: occupational physical activity level; TDEI: total daily energy intake.

[b] Participants' occupation were categorized into high (social classes Ⅰ–Ⅱ), medium (social classes Ⅲ–Ⅳ) and low (social class Ⅴ). Adult education was grouped into high (⩾12 years formal education), medium (8-11 years formal education), and low (<8 years formal education)

Table S18. The association between adult SEP and BMI trajectories in a large sample (Females)

	**Analysis sample（N=3, 138）**				**Sample with complete adult socioeconomic positions（N=18, 806）**			
	Progressive overweight vs. normal-stable BMI				Progressive overweight vs. normal-stable BMI			
	Adult occupational position		Adult education		Adult occupational position		Adult education	
	Lowest	Highest	Lowest	Highest	Lowest	Highest	Lowest	Highest
	OR (95% CI)	OR (95% CI)	OR (95% CI)	OR (95% CI)	OR (95% CI)	OR (95% CI)	OR (95% CI)	OR (95% CI)
Model 1	1.00(ref.)	0.57(0.36;0.89)	1.00(ref.)	0.64(0.41;1.00)	1.00(ref.)	1.11(0.97;1.26)	1.00(ref.)	1.04(0.92;1.18)
Model 2	1.00(ref.)	0.60(0.38;0.94)	1.00(ref.)	0.65(0.42;1.02)	1.00(ref.)	1.10(0.96;1.25)	1.00(ref.)	1.01(0.90;1.14)
Model 3	1.00(ref.)	0.56(0.35;0.88)	1.00(ref.)	0.63(0.40;0.98)	1.00(ref.)	1.10(0.96;1.25)	1.00(ref.)	1.00(0.88;1.12)
Model 4	1.00(ref.)	0.58(0.37;0.91)	1.00(ref.)	0.66(0.42;1.03)	1.00(ref.)	1.10(0.97;1.26)	1.00(ref.)	1.03(0.92;1.17)
Model 5	1.00(ref.)	0.59(0.37;0.94)	1.00(ref.)	0.66(0.41;1.04)	1.00(ref.)	1.09(0.95;1.24)	1.00(ref.)	0.96(0.85;1.09)
	Progressive obesity vs. normal-stable BMI				Progressive obesity vs. normal-stable BMI			
	Adult occupational position		Adult education		Adult occupational position		Adult education	
	Lowest	Highest	Lowest	Highest	Lowest	Highest	Lowest	Highest
	OR (95% CI)	OR (95% CI)	OR (95% CI)	OR (95% CI)	OR (95% CI)	OR (95% CI)	OR (95% CI)	OR (95% CI)
Model 1	1.00(ref.)	0.28(0.10;0.72)	1.00(ref.)	1.16(0.50;2.65)	1.00(ref.)	1.05(0.87;1.26)	1.00(ref.)	0.82(0.68;0.98)
Model 2	1.00(ref.)	0.27(0.10;0.70)	1.00(ref.)	1.11(0.48;2.55)	1.00(ref.)	1.05(0.87;1.27)	1.00(ref.)	0.79(0.65;0.95)
Model 3	1.00(ref.)	0.27(0.11;0.72)	1.00(ref.)	1.21(0.52;2.78)	1.00(ref.)	1.02(0.85;1.24)	1.00(ref.)	0.76(0.63;0.92)
Model 4	1.00(ref.)	0.30(0.12;0.78)	1.00(ref.)	1.18(0.52;2.70)	1.00(ref.)	1.03(0.86;1.25)	1.00(ref.)	0.80(0.66;0.96)
Model 5	1.00(ref.)	0.28(0.11;0.75)	1.00(ref.)	1.20(0.52;2.76)	1.00(ref.)	1.01(0.83;1.23)	1.00(ref.)	0.73(0.61;0.89)

[a] Model 1: Gender +residence +age; Model 2: Model 1+ change in smoking and drinking; Model 3: Model 1+ change in OPAL; Model 4: Model 1+ change in TDEI; Model 5: Model 1 + change in smoking, drinking, OPAL, and TDEI. ref: reference; OPAL: occupational physical activity level; TDEI: total daily energy intake.

[b] Participants’ occupation were categorized into high (social classes Ⅰ–Ⅱ), medium (social classes Ⅲ–Ⅳ) and low (social class Ⅴ). Adult education was grouped into high (⩾12 years formal education), medium (8-11 years formal education), and low (<8 years formal education).

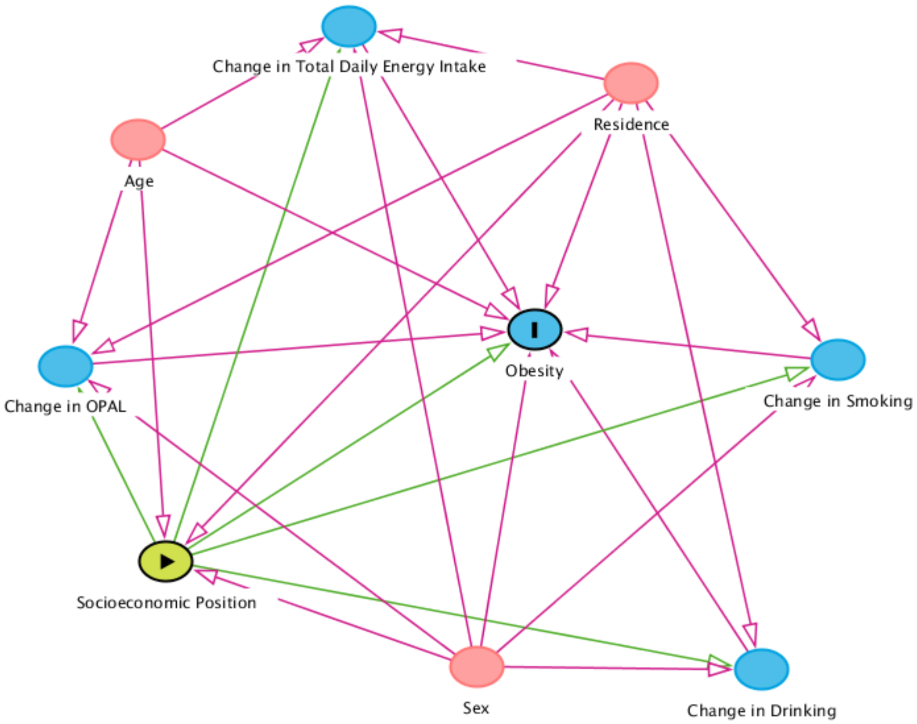

Figure S1. Theoretical model of the association between SEP and obesity based on a directed acyclic graph (DAG)

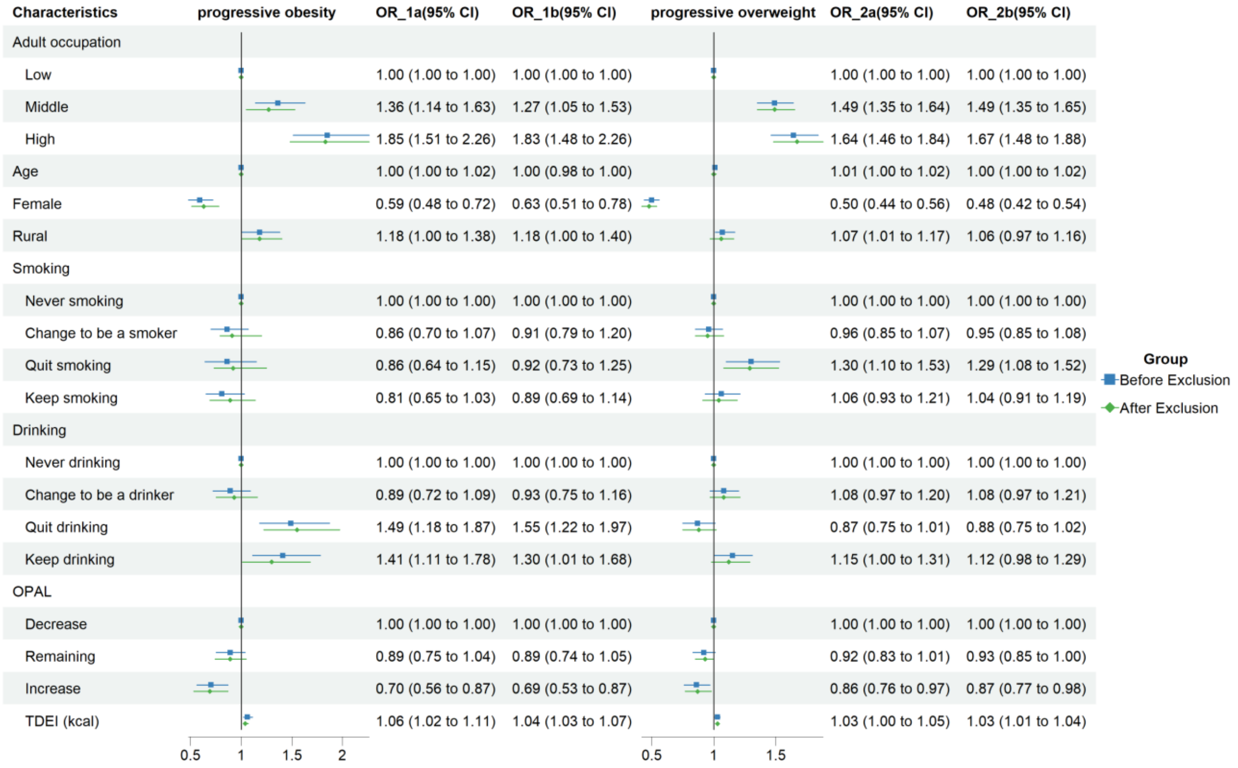

Figure S2. **The association between adult occupation and BMI trajectories (before & after exclude chronic diseases)**

OR_1a: “progressive obesity” trajectory before excluding chronic diseases; OR_1b: “progressive obesity” trajectory after excluding chronic diseases; OR_2a: “progressive overweight” trajectory before excluding chronic diseases; OR_2b: “progressive overweight” trajectory after excluding chronic diseases; OPAL: Physical Activity Level； TDEI: total daily energy intake.

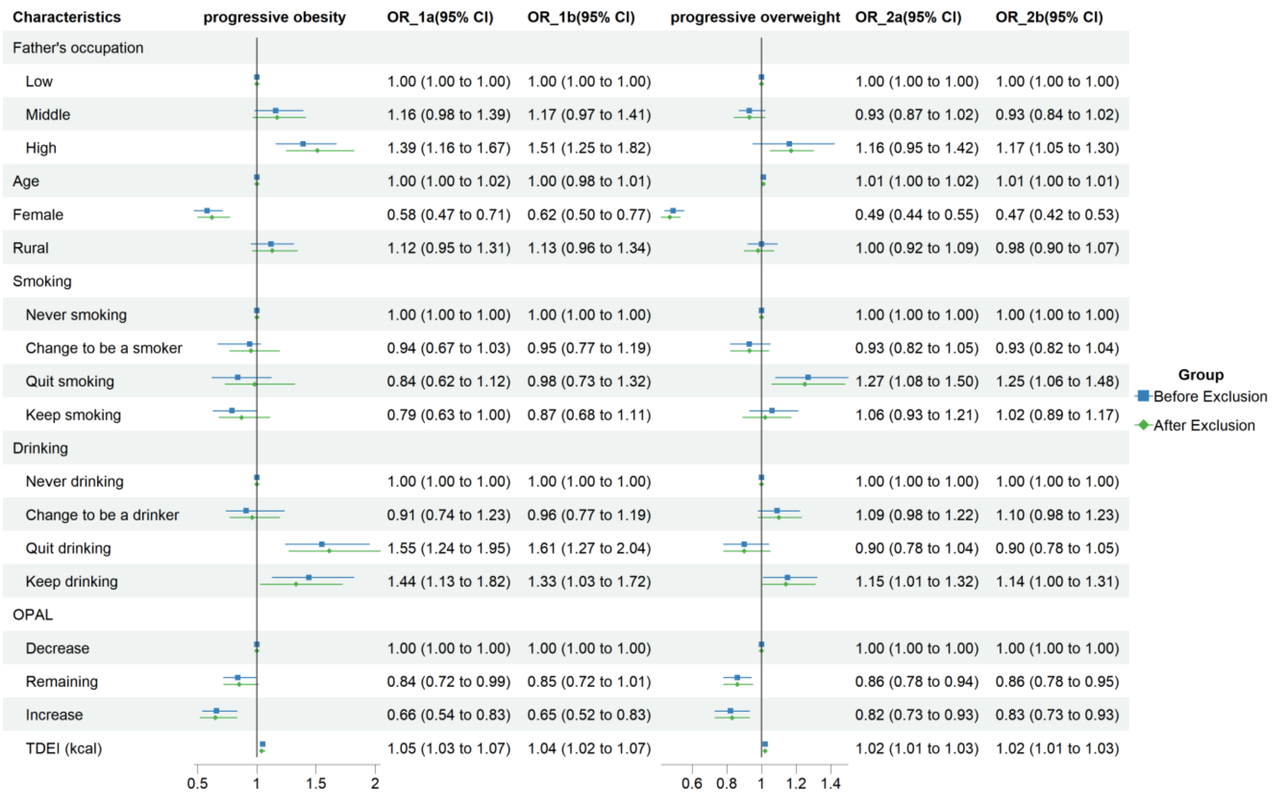

Figure S3**. The association between father's occupation and BMI trajectories (before & after exclude chronic diseases)**

OR_1a: "progressive obesity" trajectory before excluding chronic diseases; OR_1b: "progressive obesity" trajectory after excluding chronic diseases; OR_2a: "progressive overweight" trajectory before excluding chronic diseases; OR_2b: "progressive overweight" trajectory after excluding chronic diseases; OPAL: Physical Activity Level； TDEI: total daily energy intake.

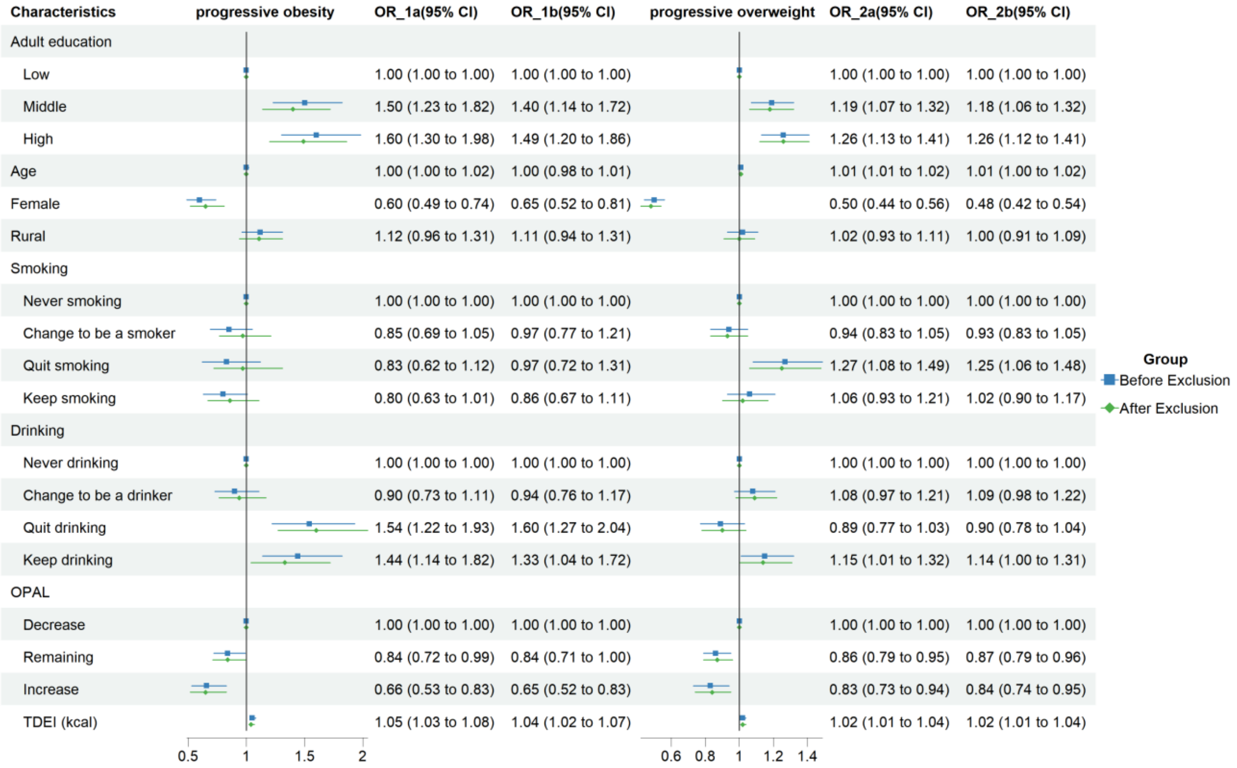

Characteristics	OR_1a(95% CI)	OR_1b(95% CI)	OR_2a(95% CI)	OR_2b(95% CI)
Adult education				
Low	1.00 (1.00 to 1.00)	1.00 (1.00 to 1.00)	1.00 (1.00 to 1.00)	1.00 (1.00 to 1.00)
Middle	1.50 (1.23 to 1.82)	1.40 (1.14 to 1.72)	1.19 (1.07 to 1.32)	1.18 (1.06 to 1.32)
High	1.60 (1.30 to 1.98)	1.49 (1.20 to 1.86)	1.26 (1.13 to 1.41)	1.26 (1.12 to 1.41)
Age	1.00 (1.00 to 1.02)	1.00 (0.98 to 1.01)	1.01 (1.01 to 1.02)	1.01 (1.00 to 1.02)
Female	0.60 (0.49 to 0.74)	0.65 (0.52 to 0.81)	0.50 (0.44 to 0.56)	0.48 (0.42 to 0.54)
Rural	1.12 (0.96 to 1.31)	1.11 (0.94 to 1.31)	1.02 (0.93 to 1.11)	1.00 (0.91 to 1.09)
Smoking				
Never smoking	1.00 (1.00 to 1.00)	1.00 (1.00 to 1.00)	1.00 (1.00 to 1.00)	1.00 (1.00 to 1.00)
Change to be a smoker	0.85 (0.69 to 1.05)	0.97 (0.77 to 1.21)	0.94 (0.83 to 1.05)	0.93 (0.83 to 1.05)
Quit smoking	0.83 (0.62 to 1.12)	0.97 (0.72 to 1.31)	1.27 (1.08 to 1.49)	1.25 (1.06 to 1.48)
Keep smoking	0.80 (0.63 to 1.01)	0.86 (0.67 to 1.11)	1.06 (0.93 to 1.21)	1.02 (0.90 to 1.17)
Drinking				
Never drinking	1.00 (1.00 to 1.00)	1.00 (1.00 to 1.00)	1.00 (1.00 to 1.00)	1.00 (1.00 to 1.00)
Change to be a drinker	0.90 (0.73 to 1.11)	0.94 (0.76 to 1.17)	1.08 (0.97 to 1.21)	1.09 (0.98 to 1.22)
Quit drinking	1.54 (1.22 to 1.93)	1.60 (1.27 to 2.04)	0.89 (0.77 to 1.03)	0.90 (0.78 to 1.04)
Keep drinking	1.44 (1.14 to 1.82)	1.33 (1.04 to 1.72)	1.15 (1.01 to 1.32)	1.14 (1.00 to 1.31)
OPAL				
Decrease	1.00 (1.00 to 1.00)	1.00 (1.00 to 1.00)	1.00 (1.00 to 1.00)	1.00 (1.00 to 1.00)
Remaining	0.84 (0.72 to 0.99)	0.84 (0.71 to 1.00)	0.86 (0.79 to 0.95)	0.87 (0.79 to 0.96)
Increase	0.66 (0.53 to 0.83)	0.65 (0.52 to 0.83)	0.83 (0.73 to 0.94)	0.84 (0.74 to 0.95)
TDEI (kcal)	1.05 (1.03 to 1.08)	1.04 (1.02 to 1.07)	1.02 (1.01 to 1.04)	1.02 (1.01 to 1.04)

Figure S4**. The association between adult education and BMI trajectories (before & after exclude chronic diseases)**

OR_1a: "progressive obesity" trajectory before excluding chronic diseases; OR_1b: "progressive obesity" trajectory after excluding chronic diseases; OR_2a: "progressive overweight" trajectory before excluding chronic diseases; OR_2b: "progressive overweight" trajectory after excluding chronic diseases;OPAL: Physical Activity Level； TDEI: total daily energy intake.

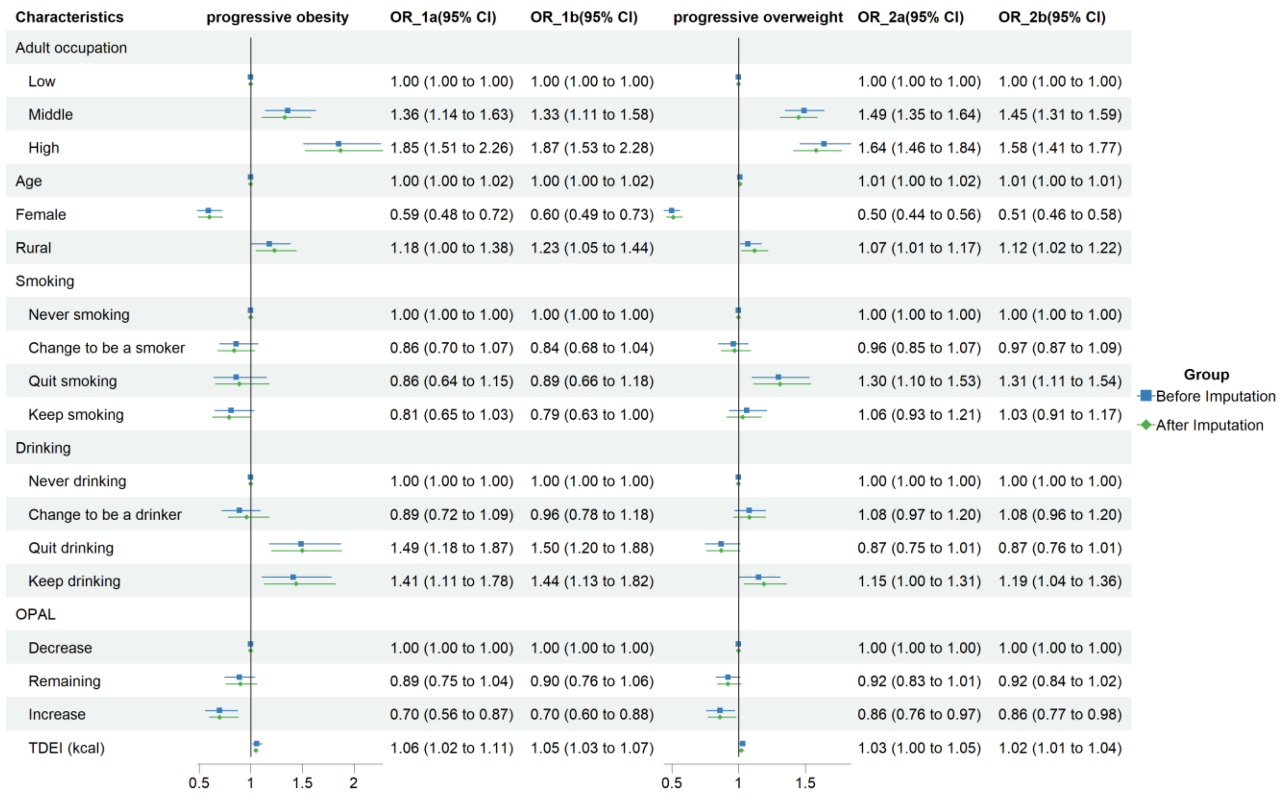

Characteristics	OR_1a(95% CI)	OR_1b(95% CI)	OR_2a(95% CI)	OR_2b(95% CI)
Adult occupation				
Low	1.00 (1.00 to 1.00)	1.00 (1.00 to 1.00)	1.00 (1.00 to 1.00)	1.00 (1.00 to 1.00)
Middle	1.36 (1.14 to 1.63)	1.33 (1.11 to 1.58)	1.49 (1.35 to 1.64)	1.45 (1.31 to 1.59)
High	1.85 (1.51 to 2.26)	1.87 (1.53 to 2.28)	1.64 (1.46 to 1.84)	1.58 (1.41 to 1.77)
Age	1.00 (1.00 to 1.02)	1.00 (1.00 to 1.02)	1.01 (1.00 to 1.02)	1.01 (1.00 to 1.01)
Female	0.59 (0.48 to 0.72)	0.60 (0.49 to 0.73)	0.50 (0.44 to 0.56)	0.51 (0.46 to 0.58)
Rural	1.18 (1.00 to 1.38)	1.23 (1.05 to 1.44)	1.07 (1.01 to 1.17)	1.12 (1.02 to 1.22)
Smoking				
Never smoking	1.00 (1.00 to 1.00)	1.00 (1.00 to 1.00)	1.00 (1.00 to 1.00)	1.00 (1.00 to 1.00)
Change to be a smoker	0.86 (0.70 to 1.07)	0.84 (0.68 to 1.04)	0.96 (0.85 to 1.07)	0.97 (0.87 to 1.09)
Quit smoking	0.86 (0.64 to 1.15)	0.89 (0.66 to 1.18)	1.30 (1.10 to 1.53)	1.31 (1.11 to 1.54)
Keep smoking	0.81 (0.65 to 1.03)	0.79 (0.63 to 1.00)	1.06 (0.93 to 1.21)	1.03 (0.91 to 1.17)
Drinking				
Never drinking	1.00 (1.00 to 1.00)	1.00 (1.00 to 1.00)	1.00 (1.00 to 1.00)	1.00 (1.00 to 1.00)
Change to be a drinker	0.89 (0.72 to 1.09)	0.96 (0.78 to 1.18)	1.08 (0.97 to 1.20)	1.08 (0.96 to 1.20)
Quit drinking	1.49 (1.18 to 1.87)	1.50 (1.20 to 1.88)	0.87 (0.75 to 1.01)	0.87 (0.76 to 1.01)
Keep drinking	1.41 (1.11 to 1.78)	1.44 (1.13 to 1.82)	1.15 (1.00 to 1.31)	1.19 (1.04 to 1.36)
OPAL				
Decrease	1.00 (1.00 to 1.00)	1.00 (1.00 to 1.00)	1.00 (1.00 to 1.00)	1.00 (1.00 to 1.00)
Remaining	0.89 (0.75 to 1.04)	0.90 (0.76 to 1.06)	0.92 (0.83 to 1.01)	0.92 (0.84 to 1.02)
Increase	0.70 (0.56 to 0.87)	0.70 (0.60 to 0.88)	0.86 (0.76 to 0.97)	0.86 (0.77 to 0.98)
TDEI (kcal)	1.06 (1.02 to 1.11)	1.05 (1.03 to 1.07)	1.03 (1.00 to 1.05)	1.02 (1.01 to 1.04)

Figure S5**. The association between adult occupation and BMI trajectories (before & after Multiple Imputation)**

OR_1a: "progressive obesity" trajectory before multiple imputation; OR_1b: "progressive obesity" trajectory after multiple imputation; OR_2a: "progressive overweight" trajectory before multiple imputation; OR_2b: "progressive overweight" trajectory after multiple imputation; OPAL: Physical Activity Level; TDEI: total daily energy intake.

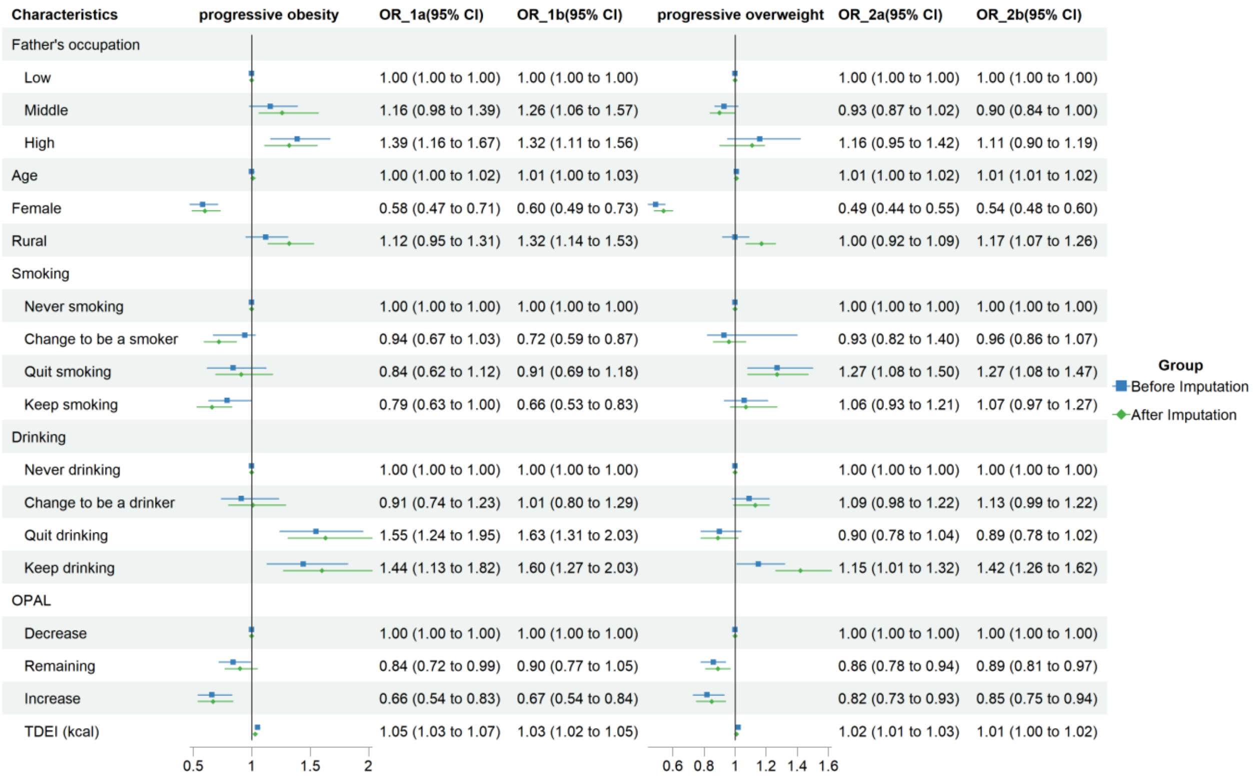

Characteristics	OR_1a(95% CI)	OR_1b(95% CI)	OR_2a(95% CI)	OR_2b(95% CI)
Father's occupation				
Low	1.00 (1.00 to 1.00)	1.00 (1.00 to 1.00)	1.00 (1.00 to 1.00)	1.00 (1.00 to 1.00)
Middle	1.16 (0.98 to 1.39)	1.26 (1.06 to 1.57)	0.93 (0.87 to 1.02)	0.90 (0.84 to 1.00)
High	1.39 (1.16 to 1.67)	1.32 (1.11 to 1.56)	1.16 (0.95 to 1.42)	1.11 (0.90 to 1.19)
Age	1.00 (1.00 to 1.02)	1.01 (1.00 to 1.03)	1.01 (1.00 to 1.02)	1.01 (1.01 to 1.02)
Female	0.58 (0.47 to 0.71)	0.60 (0.49 to 0.73)	0.49 (0.44 to 0.55)	0.54 (0.48 to 0.60)
Rural	1.12 (0.95 to 1.31)	1.32 (1.14 to 1.53)	1.00 (0.92 to 1.09)	1.17 (1.07 to 1.26)
Smoking				
Never smoking	1.00 (1.00 to 1.00)	1.00 (1.00 to 1.00)	1.00 (1.00 to 1.00)	1.00 (1.00 to 1.00)
Change to be a smoker	0.94 (0.67 to 1.03)	0.72 (0.59 to 0.87)	0.93 (0.82 to 1.40)	0.96 (0.86 to 1.07)
Quit smoking	0.84 (0.62 to 1.12)	0.91 (0.69 to 1.18)	1.27 (1.08 to 1.50)	1.27 (1.08 to 1.47)
Keep smoking	0.79 (0.63 to 1.00)	0.66 (0.53 to 0.83)	1.06 (0.93 to 1.21)	1.07 (0.97 to 1.27)
Drinking				
Never drinking	1.00 (1.00 to 1.00)	1.00 (1.00 to 1.00)	1.00 (1.00 to 1.00)	1.00 (1.00 to 1.00)
Change to be a drinker	0.91 (0.74 to 1.23)	1.01 (0.80 to 1.29)	1.09 (0.98 to 1.22)	1.13 (0.99 to 1.22)
Quit drinking	1.55 (1.24 to 1.95)	1.63 (1.31 to 2.03)	0.90 (0.78 to 1.04)	0.89 (0.78 to 1.02)
Keep drinking	1.44 (1.13 to 1.82)	1.60 (1.27 to 2.03)	1.15 (1.01 to 1.32)	1.42 (1.26 to 1.62)
OPAL				
Decrease	1.00 (1.00 to 1.00)	1.00 (1.00 to 1.00)	1.00 (1.00 to 1.00)	1.00 (1.00 to 1.00)
Remaining	0.84 (0.72 to 0.99)	0.90 (0.77 to 1.05)	0.86 (0.78 to 0.94)	0.89 (0.81 to 0.97)
Increase	0.66 (0.54 to 0.83)	0.67 (0.54 to 0.84)	0.82 (0.73 to 0.93)	0.85 (0.75 to 0.94)
TDEI (kcal)	1.05 (1.03 to 1.07)	1.03 (1.02 to 1.05)	1.02 (1.01 to 1.03)	1.01 (1.00 to 1.02)

Figure S6**. The association between father's occupation and BMI trajectories (before & after Multiple Imputation)**

OR_1a: "progressive obesity" trajectory before multiple imputation; OR_1b: "progressive obesity" trajectory after multiple imputation; OR_2a: "progressive overweight" trajectory before multiple imputation; OR_2b: "progressive overweight" trajectory after multiple imputation; OPAL: Physical Activity Level; TDEI: total daily energy intake.

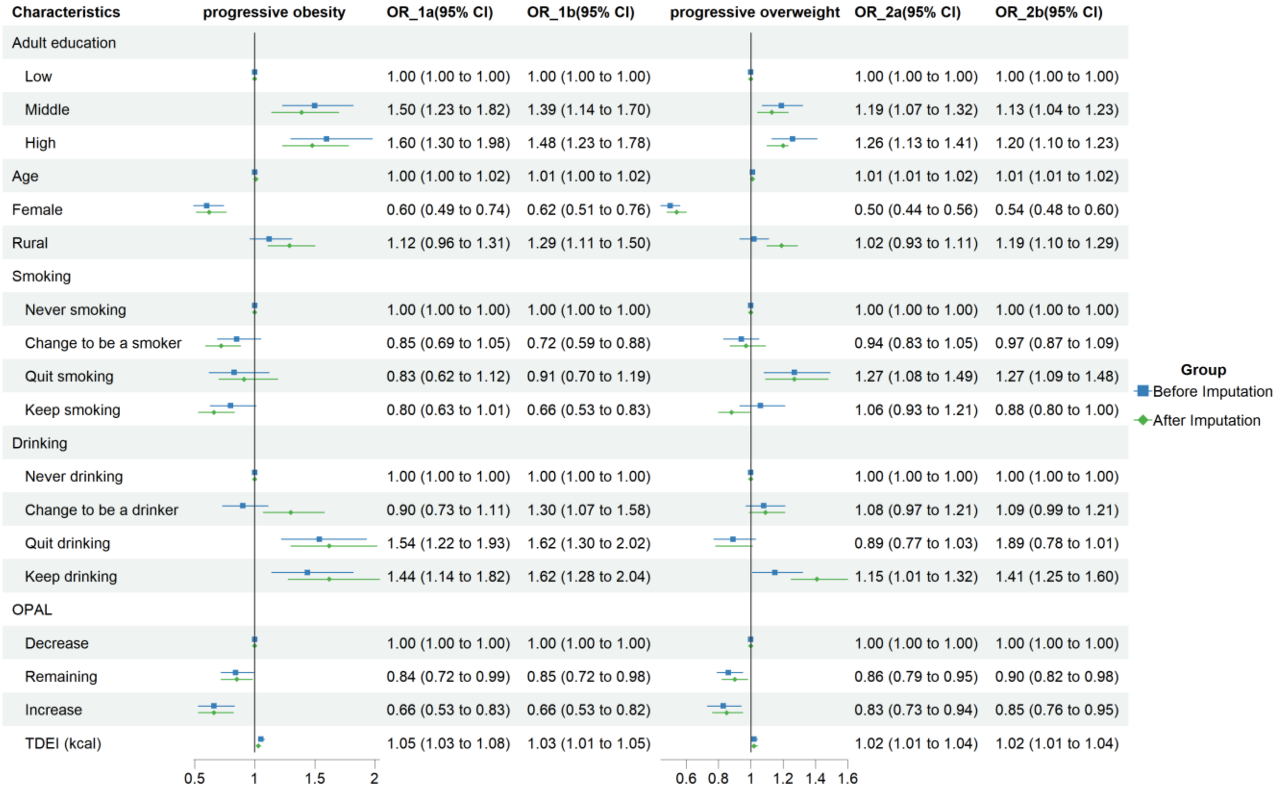

Characteristics	OR_1a(95% CI)	OR_1b(95% CI)	OR_2a(95% CI)	OR_2b(95% CI)
Adult education				
Low	1.00 (1.00 to 1.00)	1.00 (1.00 to 1.00)	1.00 (1.00 to 1.00)	1.00 (1.00 to 1.00)
Middle	1.50 (1.23 to 1.82)	1.39 (1.14 to 1.70)	1.19 (1.07 to 1.32)	1.13 (1.04 to 1.23)
High	1.60 (1.30 to 1.98)	1.48 (1.23 to 1.78)	1.26 (1.13 to 1.41)	1.20 (1.10 to 1.23)
Age	1.00 (1.00 to 1.02)	1.01 (1.00 to 1.02)	1.01 (1.01 to 1.02)	1.01 (1.01 to 1.02)
Female	0.60 (0.49 to 0.74)	0.62 (0.51 to 0.76)	0.50 (0.44 to 0.56)	0.54 (0.48 to 0.60)
Rural	1.12 (0.96 to 1.31)	1.29 (1.11 to 1.50)	1.02 (0.93 to 1.11)	1.19 (1.10 to 1.29)
Smoking				
Never smoking	1.00 (1.00 to 1.00)	1.00 (1.00 to 1.00)	1.00 (1.00 to 1.00)	1.00 (1.00 to 1.00)
Change to be a smoker	0.85 (0.69 to 1.05)	0.72 (0.59 to 0.88)	0.94 (0.83 to 1.05)	0.97 (0.87 to 1.09)
Quit smoking	0.83 (0.62 to 1.12)	0.91 (0.70 to 1.19)	1.27 (1.08 to 1.49)	1.27 (1.09 to 1.48)
Keep smoking	0.80 (0.63 to 1.01)	0.66 (0.53 to 0.83)	1.06 (0.93 to 1.21)	0.88 (0.80 to 1.00)
Drinking				
Never drinking	1.00 (1.00 to 1.00)	1.00 (1.00 to 1.00)	1.00 (1.00 to 1.00)	1.00 (1.00 to 1.00)
Change to be a drinker	0.90 (0.73 to 1.11)	1.30 (1.07 to 1.58)	1.08 (0.97 to 1.21)	1.09 (0.99 to 1.21)
Quit drinking	1.54 (1.22 to 1.93)	1.62 (1.30 to 2.02)	0.89 (0.77 to 1.03)	1.89 (0.78 to 1.01)
Keep drinking	1.44 (1.14 to 1.82)	1.62 (1.28 to 2.04)	1.15 (1.01 to 1.32)	1.41 (1.25 to 1.60)
OPAL				
Decrease	1.00 (1.00 to 1.00)	1.00 (1.00 to 1.00)	1.00 (1.00 to 1.00)	1.00 (1.00 to 1.00)
Remaining	0.84 (0.72 to 0.99)	0.85 (0.72 to 0.98)	0.86 (0.79 to 0.95)	0.90 (0.82 to 0.98)
Increase	0.66 (0.53 to 0.83)	0.66 (0.53 to 0.82)	0.83 (0.73 to 0.94)	0.85 (0.76 to 0.95)
TDEI (kcal)	1.05 (1.03 to 1.08)	1.03 (1.01 to 1.05)	1.02 (1.01 to 1.04)	1.02 (1.01 to 1.04)

Figure S7. **The association between adult education and BMI trajectories (before & after Multiple Imputation)**

OR_1a: “progressive obesity” trajectory before multiple imputation; OR_1b: “progressive obesity” trajectory after multiple imputation; OR_2a: “progressive overweight” trajectory before multiple imputation; OR_2b: “progressive overweight” trajectory after multiple imputation; OPAL: Physical Activity Level； TDEI: total daily energy intake.

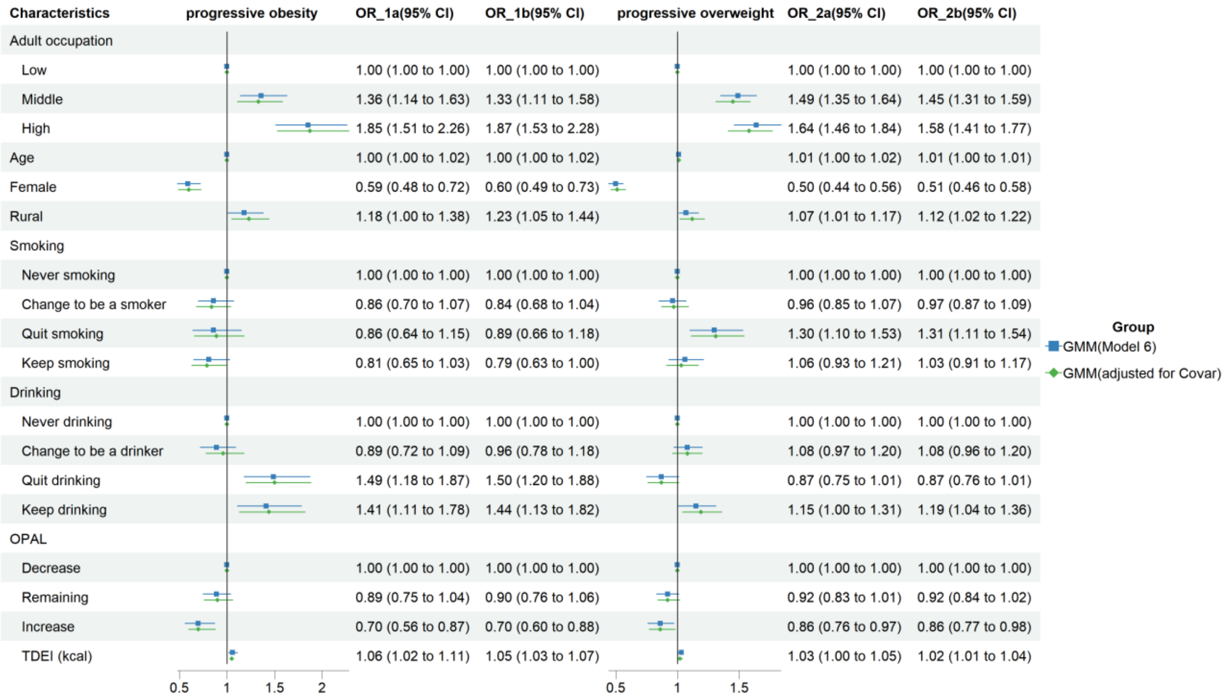

Characteristics	OR_1a(95% CI)	OR_1b(95% CI)	OR_2a(95% CI)	OR_2b(95% CI)
Adult occupation				
Low	1.00 (1.00 to 1.00)	1.00 (1.00 to 1.00)	1.00 (1.00 to 1.00)	1.00 (1.00 to 1.00)
Middle	1.36 (1.14 to 1.63)	1.33 (1.11 to 1.58)	1.49 (1.35 to 1.64)	1.45 (1.31 to 1.59)
High	1.85 (1.51 to 2.26)	1.87 (1.53 to 2.28)	1.64 (1.46 to 1.84)	1.58 (1.41 to 1.77)
Age	1.00 (1.00 to 1.02)	1.00 (1.00 to 1.02)	1.01 (1.00 to 1.02)	1.01 (1.00 to 1.01)
Female	0.59 (0.48 to 0.72)	0.60 (0.49 to 0.73)	0.50 (0.44 to 0.56)	0.51 (0.46 to 0.58)
Rural	1.18 (1.00 to 1.38)	1.23 (1.05 to 1.44)	1.07 (1.01 to 1.17)	1.12 (1.02 to 1.22)
Smoking				
Never smoking	1.00 (1.00 to 1.00)	1.00 (1.00 to 1.00)	1.00 (1.00 to 1.00)	1.00 (1.00 to 1.00)
Change to be a smoker	0.86 (0.70 to 1.07)	0.84 (0.68 to 1.04)	0.96 (0.85 to 1.07)	0.97 (0.87 to 1.09)
Quit smoking	0.86 (0.64 to 1.15)	0.89 (0.66 to 1.18)	1.30 (1.10 to 1.53)	1.31 (1.11 to 1.54)
Keep smoking	0.81 (0.65 to 1.03)	0.79 (0.63 to 1.00)	1.06 (0.93 to 1.21)	1.03 (0.91 to 1.17)
Drinking				
Never drinking	1.00 (1.00 to 1.00)	1.00 (1.00 to 1.00)	1.00 (1.00 to 1.00)	1.00 (1.00 to 1.00)
Change to be a drinker	0.89 (0.72 to 1.09)	0.96 (0.78 to 1.18)	1.08 (0.97 to 1.20)	1.08 (0.96 to 1.20)
Quit drinking	1.49 (1.18 to 1.87)	1.50 (1.20 to 1.88)	0.87 (0.75 to 1.01)	0.87 (0.76 to 1.01)
Keep drinking	1.41 (1.11 to 1.78)	1.44 (1.13 to 1.82)	1.15 (1.00 to 1.31)	1.19 (1.04 to 1.36)
OPAL				
Decrease	1.00 (1.00 to 1.00)	1.00 (1.00 to 1.00)	1.00 (1.00 to 1.00)	1.00 (1.00 to 1.00)
Remaining	0.89 (0.75 to 1.04)	0.90 (0.76 to 1.06)	0.92 (0.83 to 1.01)	0.92 (0.84 to 1.02)
Increase	0.70 (0.56 to 0.87)	0.70 (0.60 to 0.88)	0.86 (0.76 to 0.97)	0.86 (0.77 to 0.98)
TDEI (kcal)	1.06 (1.02 to 1.11)	1.05 (1.03 to 1.07)	1.03 (1.00 to 1.05)	1.02 (1.01 to 1.04)

Figure S8**. The association between adult occupation and BMI trajectories (GMM & GMM adjusted for the covariates)**

OR_1a: "progressive obesity" trajectory before multiple imputation; OR_1b: "progressive obesity" trajectory after multiple imputation; OR_2a: "progressive overweight" trajectory before multiple imputation; OR_2b: "progressive overweight" trajectory after multiple imputation; OPAL: Physical Activity Level; TDEI: total daily energy intake.

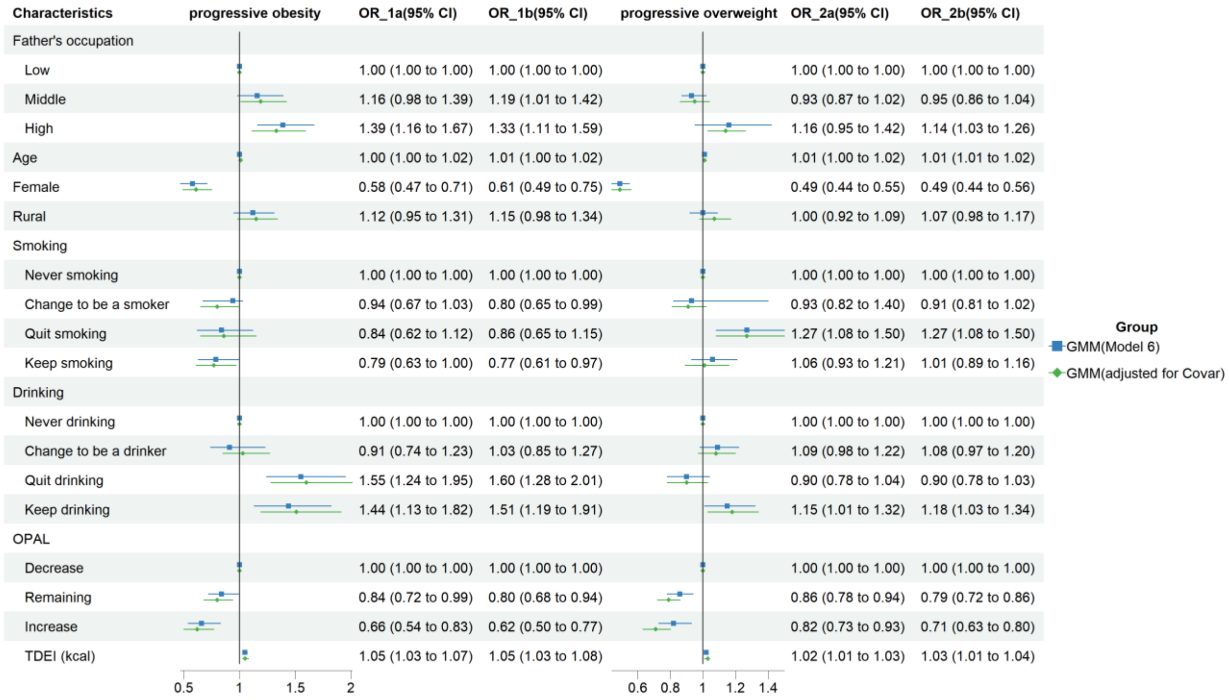

Figure S9. **The association between father's occupation and BMI trajectories (GMM & GMM adjusted for the covariates)**

OR_1a: "progressive obesity" trajectory before multiple imputation; OR_1b: "progressive obesity" trajectory after multiple imputation; OR_2a: "progressive overweight" trajectory before multiple imputation; OR_2b: "progressive overweight" trajectory after multiple imputation; OPAL: Physical Activity Level; TDEI: total daily energy intake.

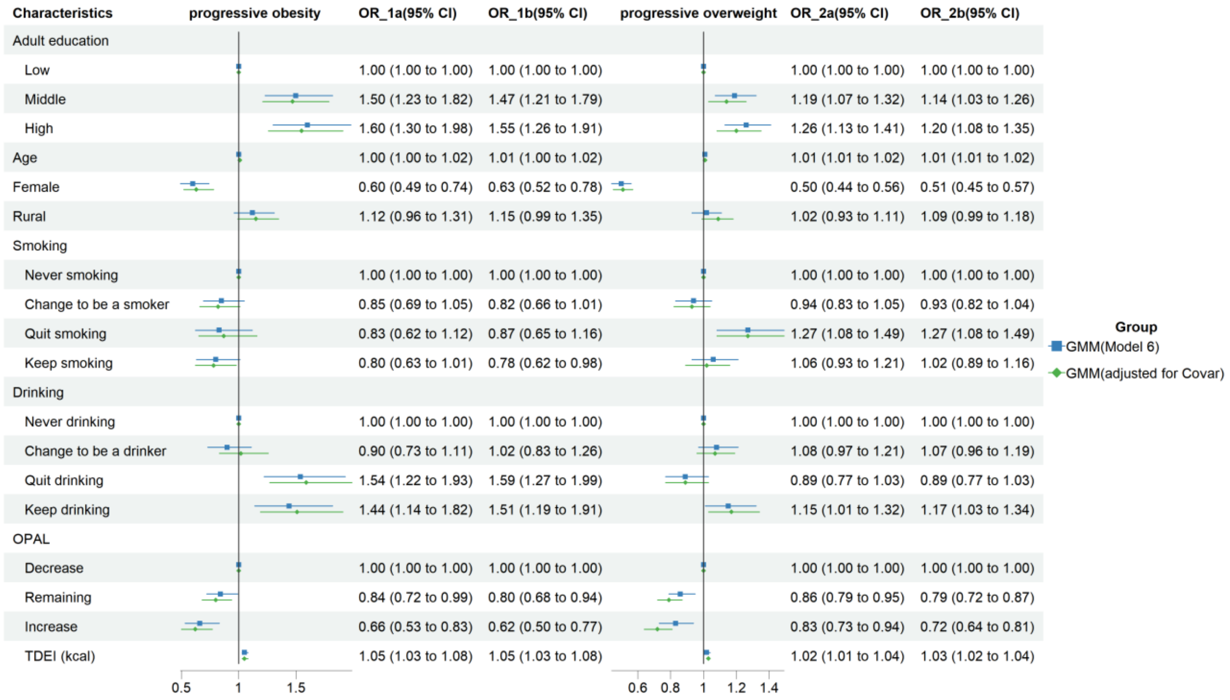

Characteristics	OR_1a(95% CI)	OR_1b(95% CI)	OR_2a(95% CI)	OR_2b(95% CI)
Adult education				
Low	1.00 (1.00 to 1.00)	1.00 (1.00 to 1.00)	1.00 (1.00 to 1.00)	1.00 (1.00 to 1.00)
Middle	1.50 (1.23 to 1.82)	1.47 (1.21 to 1.79)	1.19 (1.07 to 1.32)	1.14 (1.03 to 1.26)
High	1.60 (1.30 to 1.98)	1.55 (1.26 to 1.91)	1.26 (1.13 to 1.41)	1.20 (1.08 to 1.35)
Age	1.00 (1.00 to 1.02)	1.01 (1.00 to 1.02)	1.01 (1.01 to 1.02)	1.01 (1.01 to 1.02)
Female	0.60 (0.49 to 0.74)	0.63 (0.52 to 0.78)	0.50 (0.44 to 0.56)	0.51 (0.45 to 0.57)
Rural	1.12 (0.96 to 1.31)	1.15 (0.99 to 1.35)	1.02 (0.93 to 1.11)	1.09 (0.99 to 1.18)
Smoking				
Never smoking	1.00 (1.00 to 1.00)	1.00 (1.00 to 1.00)	1.00 (1.00 to 1.00)	1.00 (1.00 to 1.00)
Change to be a smoker	0.85 (0.69 to 1.05)	0.82 (0.66 to 1.01)	0.94 (0.83 to 1.05)	0.93 (0.82 to 1.04)
Quit smoking	0.83 (0.62 to 1.12)	0.87 (0.65 to 1.16)	1.27 (1.08 to 1.49)	1.27 (1.08 to 1.49)
Keep smoking	0.80 (0.63 to 1.01)	0.78 (0.62 to 0.98)	1.06 (0.93 to 1.21)	1.02 (0.89 to 1.16)
Drinking				
Never drinking	1.00 (1.00 to 1.00)	1.00 (1.00 to 1.00)	1.00 (1.00 to 1.00)	1.00 (1.00 to 1.00)
Change to be a drinker	0.90 (0.73 to 1.11)	1.02 (0.83 to 1.26)	1.08 (0.97 to 1.21)	1.07 (0.96 to 1.19)
Quit drinking	1.54 (1.22 to 1.93)	1.59 (1.27 to 1.99)	0.89 (0.77 to 1.03)	0.89 (0.77 to 1.03)
Keep drinking	1.44 (1.14 to 1.82)	1.51 (1.19 to 1.91)	1.15 (1.01 to 1.32)	1.17 (1.03 to 1.34)
OPAL				
Decrease	1.00 (1.00 to 1.00)	1.00 (1.00 to 1.00)	1.00 (1.00 to 1.00)	1.00 (1.00 to 1.00)
Remaining	0.84 (0.72 to 0.99)	0.80 (0.68 to 0.94)	0.86 (0.79 to 0.95)	0.79 (0.72 to 0.87)
Increase	0.66 (0.53 to 0.83)	0.62 (0.50 to 0.77)	0.83 (0.73 to 0.94)	0.72 (0.64 to 0.81)
TDEI (kcal)	1.05 (1.03 to 1.08)	1.05 (1.03 to 1.08)	1.02 (1.01 to 1.04)	1.03 (1.02 to 1.04)

Figure S10**. The association between adult education and BMI trajectories (GMM & GMM adjusted for the covariates)**

OR_1a: “progressive obesity” trajectory before multiple imputation**;** OR_1b: “progressive obesity” trajectory after multiple imputation**;** OR_2a: “progressive overweight” trajectory before multiple imputation**;** OR_2b: “progressive overweight” trajectory after multiple imputation**;** OPAL: Physical Activity Level； TDEI: total daily energy intake.

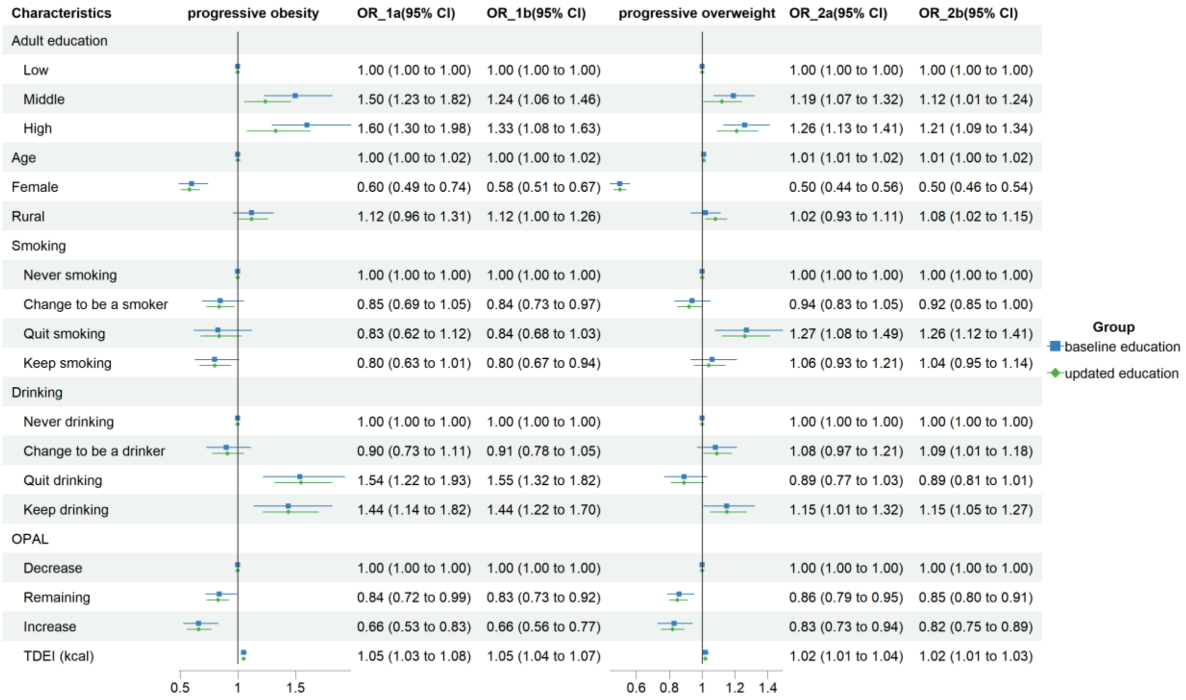

Characteristics	OR_1a(95% CI)	OR_1b(95% CI)	OR_2a(95% CI)	OR_2b(95% CI)
Adult education				
Low	1.00 (1.00 to 1.00)	1.00 (1.00 to 1.00)	1.00 (1.00 to 1.00)	1.00 (1.00 to 1.00)
Middle	1.50 (1.23 to 1.82)	1.24 (1.06 to 1.46)	1.19 (1.07 to 1.32)	1.12 (1.01 to 1.24)
High	1.60 (1.30 to 1.98)	1.33 (1.08 to 1.63)	1.26 (1.13 to 1.41)	1.21 (1.09 to 1.34)
Age	1.00 (1.00 to 1.02)	1.00 (1.00 to 1.02)	1.01 (1.01 to 1.02)	1.01 (1.00 to 1.02)
Female	0.60 (0.49 to 0.74)	0.58 (0.51 to 0.67)	0.50 (0.44 to 0.56)	0.50 (0.46 to 0.54)
Rural	1.12 (0.96 to 1.31)	1.12 (1.00 to 1.26)	1.02 (0.93 to 1.11)	1.08 (1.02 to 1.15)
Smoking				
Never smoking	1.00 (1.00 to 1.00)	1.00 (1.00 to 1.00)	1.00 (1.00 to 1.00)	1.00 (1.00 to 1.00)
Change to be a smoker	0.85 (0.69 to 1.05)	0.84 (0.73 to 0.97)	0.94 (0.83 to 1.05)	0.92 (0.85 to 1.00)
Quit smoking	0.83 (0.62 to 1.12)	0.84 (0.68 to 1.03)	1.27 (1.08 to 1.49)	1.26 (1.12 to 1.41)
Keep smoking	0.80 (0.63 to 1.01)	0.80 (0.67 to 0.94)	1.06 (0.93 to 1.21)	1.04 (0.95 to 1.14)
Drinking				
Never drinking	1.00 (1.00 to 1.00)	1.00 (1.00 to 1.00)	1.00 (1.00 to 1.00)	1.00 (1.00 to 1.00)
Change to be a drinker	0.90 (0.73 to 1.11)	0.91 (0.78 to 1.05)	1.08 (0.97 to 1.21)	1.09 (1.01 to 1.18)
Quit drinking	1.54 (1.22 to 1.93)	1.55 (1.32 to 1.82)	0.89 (0.77 to 1.03)	0.89 (0.81 to 1.01)
Keep drinking	1.44 (1.14 to 1.82)	1.44 (1.22 to 1.70)	1.15 (1.01 to 1.32)	1.15 (1.05 to 1.27)
OPAL				
Decrease	1.00 (1.00 to 1.00)	1.00 (1.00 to 1.00)	1.00 (1.00 to 1.00)	1.00 (1.00 to 1.00)
Remaining	0.84 (0.72 to 0.99)	0.83 (0.73 to 0.92)	0.86 (0.79 to 0.95)	0.85 (0.80 to 0.91)
Increase	0.66 (0.53 to 0.83)	0.66 (0.56 to 0.77)	0.83 (0.73 to 0.94)	0.82 (0.75 to 0.89)
TDEI (kcal)	1.05 (1.03 to 1.08)	1.05 (1.04 to 1.07)	1.02 (1.01 to 1.04)	1.02 (1.01 to 1.03)

Figure S11**. The association between adult education and BMI trajectories (Baseline & updated education)**

OR_1a: “progressive obesity” trajectory before multiple imputation; OR_1b: “progressive obesity” trajectory after multiple imputation; OR_2a: “progressive overweight” trajectory before multiple imputation; OR_2b: “progressive overweight” trajectory after multiple imputation; OPAL: Physical Activity Level； TDEI: total daily energy intake.
